# Jumping on the moon as a potential exercise countermeasure

**DOI:** 10.1113/EP092155

**Published:** 2025-05-11

**Authors:** Patrick Swain, Filipa Santos, Luke Hughes, Dan Gordon, Nick Caplan

**Affiliations:** ^1^ Aerospace Medicine and Rehabilitation Laboratory, Faculty of Health and Life Sciences, Department of Sport, Exercise, and Rehabilitation Northumbria University Newcastle Upon‐Tyne UK; ^2^ Departamento de Física, Faculdade de Ciências Universidade de Lisboa Lisboa Portugal; ^3^ Psychology, Sport and Sensory Science, Faculty of Science and Engineering Anglia Ruskin University Cambridge UK

**Keywords:** astronaut, countermeasure, countermovement, exercise, jumping, Mars, Moon

## Abstract

The Moon's gravitational field strength (17% Earth's gravity) may facilitate the use of bodyweight jumping as an exercise countermeasure against musculoskeletal and cardiovascular deconditioning in reduced gravity settings. The present study characterised the acute physiological and kinetic responses to bodyweight jumping in simulated Lunar gravity. Nineteen healthy adults (age: 25 ± 7 years, weight: 73 ± 11 kg; height: 1.81 ± 0.05 m, ${\dot{V}}_{O_{2}\max}$: 50 ± 11 mL kg^−1^ min^−1^) performed an incremental jumping test in simulated Lunar gravity (9.5° head‐up tilt suspension) comprising 4‐min stages of jumping with 1‐min rests, beginning at 30 cm and increasing 5 cm per stage up to 70 cm. A graded exercise test (GXT) to volitional exhaustion was subsequently performed using upright cycle ergometry. Cardiorespiratory outcomes (${\dot{V}}_{O_{2}}$, ${\dot{V}}_{CO_{2}}$, ${\dot{V}}_{E}$, breathing frequency, respiratory exchange ratio and heart rate (HR)) and peak vertical ground reaction forces (vGRF) increased linearly (*R* ^2^ = 0.77–0.97) and blood lactate concentrations increased exponentially with jump height (*R* ^2^ = 0.98). Participants achieved HRs of 158 ± 17 beats min^−1^ (88 ± 9% HR_max_), metabolic rates of 35 ± 6 mL kg^−1^ min^−1^ (71 ± 9% ${\dot{V}}_{O_{2}\max}$), blood lactate concentrations of 5.8 ± 1.7 mmol L^−1^ and peak vGRFs of 119 ± 17% bodyweight. Jumping at ∼20% bodyweight requires no equipment, allows for submaximal cardiovascular exercise intensities with and without blood lactate accumulation, and may have value as an exercise countermeasure in Lunar/Martian surface habitats.

## Abstract (highlights)

- **What is the central question of this study?**
  What exercise intensities, blood lactate concentrations, and vertical ground reaction forces can be achieved during bodyweight jumping in simulated Lunar gravity?
- **What is the main finding and its importance?**
  Oxygen consumption and heart rate increased linearly with jump height up to 71 ± 9% maximal oxygen consumption and 88 ± 9% heart rate maximum. Blood lactate concentrations increased in a curvilinear manner up to 5.8 ± 1.7 mmol L^−1^. Peak vertical ground reaction forces increased linearly up to ∼120 ± 20% bodyweight. Jumping at ∼20% bodyweight, therefore, can permit cardiovascular exercise in the moderate‐ and heavy‐intensity domains that may benefit cardiovascular and musculoskeletal health.

## 1 INTRODUCTION

Human space exploration missions to the Moon (17% Earth's gravity) and Mars (38% Earth's gravity) will challenge astronaut health and performance due to the physiological consequences of living and working in micro‐ and hypo‐gravity environments. Exposure to microgravity during spaceflight causes significant, and often progressive, deconditioning of the musculoskeletal, cardiovascular, and sensorimotor systems (Comfort et al., 2021; LeBlanc et al., 1995; LeBlanc et al., 2000; Lee et al., 2015; Lee et al., 2010; Scott et al., 2023; Stavnichuk et al., 2020; Tays et al., 2021). The effects of hypogravity exposure are relatively less understood; however, a growing body of evidence supports the hypothesis that Lunar and Martian gravities will be insufficient to prevent deconditioning (Cavanagh et al., 2013; Ko et al., 2020; Mortreux et al., 2019; Richter et al., 2017; Swain et al., 2022b; Swain et al., 2022a). Protection against micro‐ and hypo‐gravity‐induced deconditioning via exercise countermeasures will, therefore, be critical to maintaining crew autonomy, operational performance, and mitigating adverse risks.

Upholding the physical capability of crewmembers during surface exploration missions is essential, as these missions may require astronauts to perform extra‐vehicular activities (EVAs) up to 24 h per week in 4–8‐h sessions (NASA, 2022). Ground‐based EVA and emergency procedure mock‐ups have identified significant associations between human performance parameters (e.g., aerobic capacity, speed at ${\dot{V}}_{O_{2}\max}$, critical power/speed, and arm cranking and rowing peak power output) and task completion time (Ade et al., 2014; Ade et al., 2015; Alexander et al., 2019). These include activities such as a 10 km ‘walk back’, simulating a return to base following rover failure, emergency capsule egress, physical abilities field test, material transport, unloading of geological samples, equipment set‐up and operating controls/valves (Ade et al., 2014; Ade et al., 2015; Alexander et al., 2019). Ade and colleagues observed during a simulated Martian field test that task finishers had higher cardiovascular fitness levels than non‐finishers (46 ± 4 mL kg^−1^ min^−1^ vs. 37 ± 7 mL kg^−1^ min^−1^) and operated at lower fractional utilisation of aerobic power (%${\dot{V}}_{O_{2}\max}$) (65 ± 10% vs. 78 ± 5%) and predicted, via logistic regression, that the ${\dot{V}}_{O_{2}\max}$ associated with a 75% probability of task completion was 40 mL kg^−1^ min^−1^ (Ade et al., 2016). Loss of cardiovascular fitness in‐mission may compromise even the most fundamental tasks, such as suited ambulation in Lunar and Martian gravities, where metabolic rates of ∼27–38 mL kg^−1^ min^−1^ have been estimated in users performing real‐time navigation in desert‐like terrains (Norcross et al., 2008; Norcross et al., 2009). For reference, NASA's aerobic fitness standards for micro‐ and hypo‐gravity EVAs are ≥32.9 and ≥36.5 mL kg^−1^ min^−1^, respectively, such that astronauts should theoretically be working at low metabolic rates (∼30–40% ${\dot{V}}_{O_{2}\max}$) based on the average metabolic cost of spaceflight and Lunar EVAs (Strock et al., 2023). Taken together, it is critical that the physical capabilities of crewmembers do not fall below the physical requirements of mission tasks (due to deconditioning) and are ideally maintained at such a level that astronauts can operate at sustainable work rates (30–40% ${\dot{V}}_{O_{2}\max}$) to reduce fatigue and risk of volitional exhaustion (Strock et al., 2023).

Aerobic and resistive exercise have been used by space agencies as primary countermeasures against spaceflight‐induced musculoskeletal and cardiovascular deconditioning (Hackney et al., 2015; Korth, 2015; Lee et al., 2010; Petersen et al., 2016; Yarmanova et al., 2015). Astronauts onboard the International Space Station are scheduled for exercise‐related activities 2–2.5 h per day, 6 days per week. Aerobic exercise is performed using a treadmill with a bungee‐loading system and a cycle ergometer, whilst resistance exercises (squats, deadlifts, calf raises and bench press) are performed using the Advanced Resistive Exercise Device (ARED). The effectiveness of the current spaceflight exercise countermeasure programme, however, remains a contemporary issue. Despite the substantial time commitment for in‐flight exercise, astronauts returning to Earth following long‐duration spaceflight (∼6 months) still display atrophied and weakened muscles, loss of bone mineral density, reduced cardiovascular fitness and impaired physical performance (English et al., 2020; Scott et al., 2023). International space agencies are seeking to optimise exercise countermeasures by improving both the effectiveness and the efficiency of exercise (Scott et al., 2019a; Scott et al., 2019b). Future exploration‐class missions are also expected to place a number of additional constraints upon the use of exercise, such as the rate of CO_2_, heat and moisture production, restricted operating volume, food and water availability, device maintenance/repair requirements and scheduling of work/exercise activities (Laws et al., 2020; Scott et al., 2019a). Novel exercise countermeasure strategies, therefore, are being proposed for future exploration missions, including, for example, blood flow restriction training, plyometrics and even running horizontally along the walls of surface habitats (Hughes et al., 2022; Kramer, Gollhofer et al., 2017; Maynard et al., 2018; Minetti et al., 2024; Weber et al., 2019).

The benefits of jump training as a countermeasure against disuse‐induced deconditioning were recognised during two 60‐day 6° head‐down tilt bedrest campaigns conducted in 2015–2016 at:envihab (Kramer, Kümmel et al., 2017; Kramer, Gollhofer et al., 2017). A low‐volume, high‐intensity jump sledge protocol was employed in which participants performed several sets of maximal jumps/hops with short rest intervals (30–90 s) at 80–90% bodyweight for ∼10–15 min per session (∼2–4 min of which were exercising) on 48 days of the 60‐day bedrest period (Kramer, Kümmel et al., 2017). The jump training intervention, compared to the non‐exercise control group, was highly successful at attenuating loss of lean mass in the leg (0% vs. −5%), maximal torque during knee extension (−3% vs. −41%), knee flexion (−6% vs. −16%) and plantarflexion (−8% vs. −40%), knee extension torque over 90 s (−3% vs. −42%), cardiovascular fitness (${\dot{V}}_{O_{2}\max}$) (−8% vs. −32%), peak power (−12% vs. −29%), tibial bone mineral density at 4%, 38%, 66% and 98% of tibial length (−0.5% to 0.7% vs. −2.6% to −0.6%) and prevented sensorimotor impairments that were otherwise evident in the control group for up to a month into the recovery period (Blottner et al., 2019; Kramer, Gollhofer et al., 2017; Kramer, Kümmel et al., 2017; Ritzmann et al., 2018). These findings demonstrate that jumping with maximal intent close to 100% bodyweight appears to be a highly effective method for preventing (or substantially attenuating) muscle, bone, cardiovascular and sensorimotor deconditioning simultaneously during long‐term disuse with remarkable efficiency (∼10–15 min per exercise session). The attributed protective effects of the jumping/hopping intervention included high lower limb power outputs across large ranges of motion, subjecting weight‐bearing skeletal regions to frequent bouts of high impact loads and muscle forces and eliciting high metabolic rates when performed in an interval‐style manner with short rest periods (Kramer, Gollhofer et al., 2017). It is important to note that during cyclical lower limb activities such as jumping and running, leg stiffness can modulate factors including metabolic energy expenditure and mechanical stress placed on weight‐bearing skeletal regions and lower limb muscle–tendon units, which has implications for training adaptations (Ahmadi et al., 2021; Devita & Skelly, 1992; Moore et al., 2019).

The use of jumping/hopping in hypogravity environments (e.g., Lunar and Martian surface habitats) has gained recent interest as a potential exploration‐class exercise countermeasure, as it might be possible for astronauts to jump against their own bodyweight on the Moon (0.17 g) or Mars (0.38 g) as a form of exercise that would reduce or eliminate, the need for exercise hardware. Weber and colleagues identified that hopping in simulated Lunar and Martian gravities might offer musculoskeletal health benefits, after demonstrating that peak vertical ground reaction forces (vGRF), above certain hop heights, became equivalent to or surpassed, those measured during walking/running on Earth (Weber et al., 2019).

Bodyweight jumping on Earth is metabolically demanding and recreationally active adults jumping at shallow heights (∼1–2 cm) can often reach volitional exhaustion within 15‐min of exercise (Lyons et al., 2023). On the Moon and Mars, however, the reduction in bodyweight (an 80 kg individual would weigh 30 kg on Mars and 13 kg on the Moon) could allow for jumping to be performed across a wider range of the exercise intensity continuum as a function of jump height and take‐off/landing strategy. Jumping in hypogravity, therefore, may serve as an equipment‐free yet versatile training modality. The primary aim of the present study was to quantify the cardiorespiratory and metabolic responses to incremental bodyweight jumping in Lunar gravity to understand what exercise intensities (%${\dot{V}}_{O_{2}\max}$) this form of exercise can elicit and whether jumping at increasing heights may require high enough metabolic rates sufficient to observe an exponential rise in blood lactate concentration. The secondary aims were to examine the relationship between jump height and cardiorespiratory, metabolic, kinetic and perceptual responses. The aims were to: (1) determine the relationship between baseline cardiovascular fitness (${\dot{V}}_{O_{2}\max}$), fractional utilisation of ${\dot{V}}_{O_{2}\max}$ and blood lactate concentration at each jumping stage to understand whether ‘fitter’ individuals jumped at lower relative intensities at a given jump height, (2) quantify the relationship between vertical ground reaction force measures and time of force application.

[Correction added on 4th June 2025 after first online publication: The text “(3) develop a regression model to predict the jump height required to achieve a specific exercise intensity (%${\dot{V}}_{O_{2}\max}$)” has been removed from the aims in Introduction.]

## 2 METHODS

### 2.1 Participants

Nineteen healthy adults provided written informed consent to participate in the study. Participant characteristics are displayed in Table 1. The study was performed in accordance with the 2013 *Declaration of Helsinki* (except preregistration) (WMA, 2013) and received ethical approval from the Northumbria University Faculty of Health and Life Sciences Ethics Committee (reference code: 6515). Participants were non‐smokers, injury‐free, did not have any condition that could hinder jumping ability/safety, were not known to be pregnant, had not given birth <6 months prior to testing and did not present any contraindications to exercise, as per a Physical Activity Readiness Questionnaire. There were no minimum fitness requirements to partake in the study.

**TABLE 1:** Participant characteristics.

Characteristic	Cohort (*n* = 19)
Male:female (*n*)	15:4
Age (years)	25 ± 7
Mass (kg)	73 ± 11
Height (m)	1.81 ± 0.05
Systolic blood pressure (mmHg)	127 ± 10
Diastolic blood pressure (mmHg)	76 ± 8
Resting HR (beats min^−1^)	68 ± 12
${\dot{V}}_{O_{2}\max}$ (L min^−1^)	3.6 ± 0.8
${\dot{V}}_{O_{2}\max}$ (mL kg^−1^ min^−1^)	50 ± 11

*Note*: Data are means ± standard deviation. HR, heart rate.

### 2.2 Study design

Participants visited the laboratory on a single occasion for ∼2 h. During the visit, baseline/resting measures were taken (height, body mass and resting blood pressure, heart rate (HR) and blood lactate). Participants then performed an incremental jumping protocol in simulated Lunar gravity (methods described in the Section 2.3) followed by a graded exercise test (GXT) on a cycle ergometer in 1 *g* to obtain ${\dot{V}}_{O_{2}\max}$. Participants were asked to refrain from recreational stimulants and alcohol on the day of testing, not to eat within 2 h of testing and to avoid vigorous exercise 48 h before testing. All experiments were performed in an environmentally controlled laboratory (temperature: 19.5 ± 0.6°C; relative humidity: 52 ± 7%; pressure: 1038 ± 9 hPa).

### 2.3 Hypogravity analogue

**FIGURE 1:**
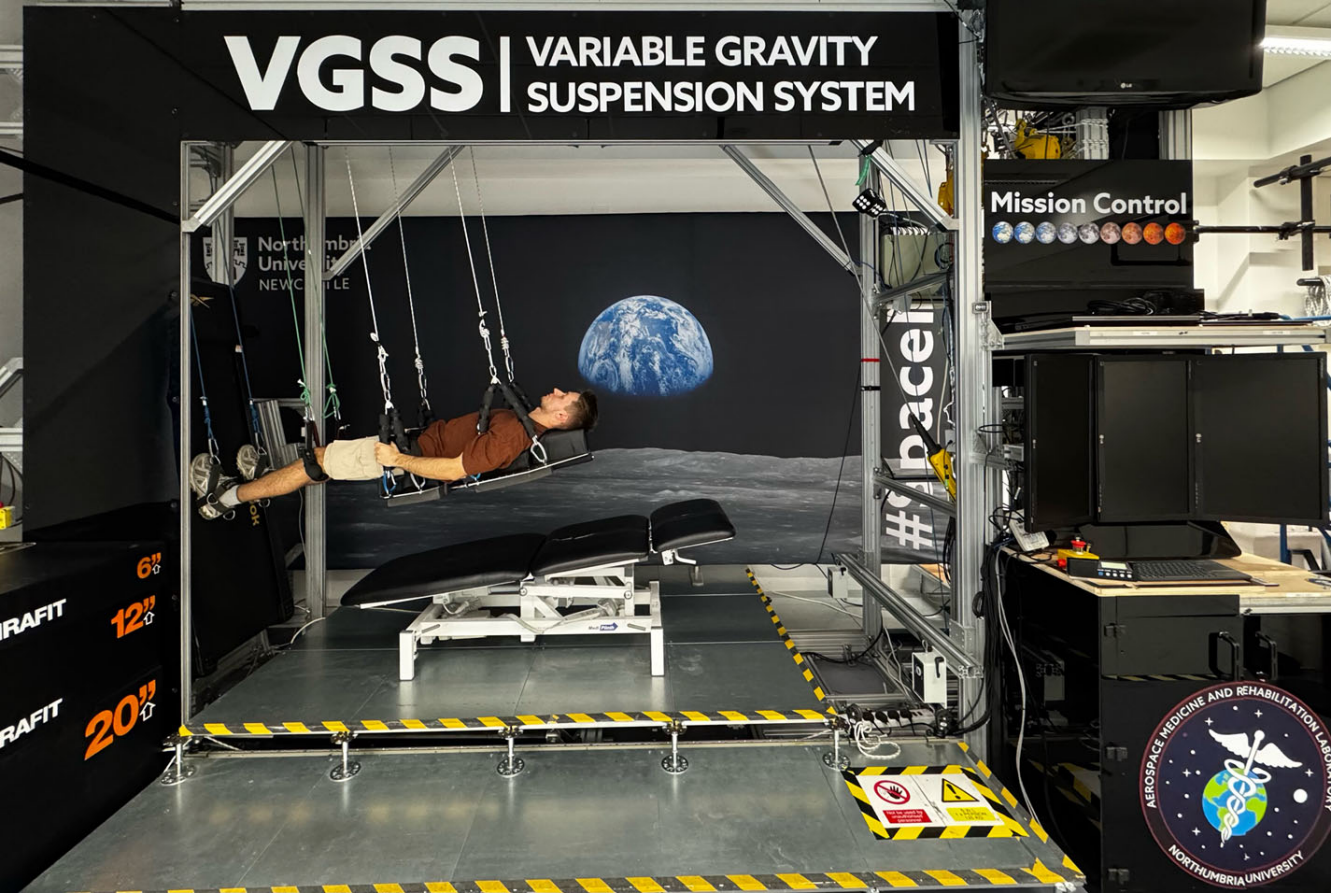
User standing in simulated Lunar gravity (9.5° head‐up tilt) within the VGSS hypogravity analogue. VGSS, Variable Gravity Suspension System.

The Variable Gravity Suspension System (VGSS) was employed to simulate Lunar gravity by suspending participants in a 9.5° head‐up tilt position to allow for ∼17% of Earth's gravity to act along the longitudinal axis of the body (*mg* sin(θ), where *m* is body mass, *g* is Earth's gravitational field strength (9.81 m s^−2^) and θ is head‐up tilt angle relative to the horizontal plane). Participants were suspended in the VGSS by a series of ropes attached to ankle and knee slings and a custom pelvic and thorax/head support plate, with the angles of each rope set to 9.5° from the vertical pane (i.e., perpendicular to the longitudinal axis of the participant's body). A treadmill (Floatride, Boston, Massachusetts, Reebok, USA) mounted onto the VGSS served as the foot platform on which participants stood and jumped. The treadmill orientation was set parallel to the suspension ropes with the belt fixed in place to prevent movement. A period of quiet standing was performed upon suspension for the measurement of ground reaction forces to verify the level of simulated hypogravity; participants experienced 17.3 ± 2.8% bodyweight loading (i.e., simulated 0.17 g). Figure 1 displays a user standing in simulated Lunar gravity within the VGSS.

### 2.4 Incremental jumping protocol

The jumping protocol comprised 4‐min stages of continuous double‐legged bodyweight countermovement jumping, starting at a jump height of 30 cm, increasing by 5 cm per stage, up to a maximum jump height of 70 cm. Jump heights >70 cm were restricted by the headroom availability of the bodyweight suspension hypogravity analogue. A 1‐min passive (standing) rest period separated each jumping stage to allow for the collection of a capillary blood sample and perceptual measures. The test was terminated when the participant could not complete a given stage due to fatigue, was unable to reach the target height or was unable to jump continuously without stopping (e.g., due to body instability). A few well‐trained participants started the protocol at 35 cm or 40 cm with the expectation that they would exhibit changes in blood lactate concentrations at higher jump heights in the same manner as if one were to decide the starting workload for a threshold exercise test. Participants were instructed to jump with relatively stiff legs and to minimise ground contact time to avoid excessive hip/knee flexion motion, a jumping technique that some individuals naturally self‐selected. No specific criteria were used to confirm or deny a ‘correct’ jumping strategy (e.g., ground contact times). Arm swinging was not permitted and participants were asked to hold onto the back support plate suspension ropes located on each side of the hip. Participants were familiarised with the jumping technique on the day of testing by jumping at heights up to that of the first stage of the incremental test until the participant was comfortable with the jumping technique, which was subsequently approved by a researcher, a process that typically took ∼5–10 min.

The displacement of the participant in line with the longitudinal axis of their body was recorded via a linear cable extension potentiometer (SP1‐50, Multicomp Pro, Leeds, UK) attached between an aluminium strut profile at the level of the foot platform and the hip region of the back‐support plate. The signal from the cable extension potentiometer was sampled by an analog to digital modular data acquisition system (NI‐9174, National Instruments, Austin, Texas, USA) via an analog input module (NI‐9201, National Instruments), at 2000 Hz and captured and stored (in.txt format) by a custom program (LabVIEW 2018, National Instruments). The cable extension potentiometer signal was zeroed to the standing position of the participant in simulated Lunar gravity across a 10‐s period. Jump height was displayed on a graph in real time on a monitor positioned directly above the participant's head in their field of view, with the target jump height (denoted by a horizontal line) also displayed. Pilot testing identified that jumping frequency could be naturally maintained at a constant rate at a given jump height within participants but differed slightly between participants (akin to running cadence). Supporting information Video S1 demonstrates a user jumping in simulated Lunar gravity within the VGSS.

### 2.5 Pulmonary gas exchange

Pulmonary gas exchange was measured on a breath‐by‐breath basis using a calibrated metabolic cart (MetaLyzer 3B, Cortex Medical, Leipzig, Germany). The following measurements were recorded: volume of oxygen consumption (${\dot{V}}_{O_{2}}$), volume of carbon dioxide production (${\dot{V}}_{CO_{2}}$), respiratory exchange ratio (RER), minute ventilation (${\dot{V}}_{E}$) and breathing frequency (BF). HR was measured via an HR monitor secured around the chest of the participant and streamed wirelessly to the metabolic cart software. The final 15‐breath average was extracted for each jumping stage (Robergs et al., 2010).

### 2.6 Blood lactate

Capillary blood samples were collected at rest and immediately following each jumping stage for the measurement of blood lactate concentration (BLA). The sampling site (fingertip or earlobe) was based on participant preference and standardised for each participant. The site was cleaned with alcohol, punctured with a lancet and encouraged to bleed, with the first droplet of blood removed and the second droplet collected in a 20 µL capillary tube and slowly mixed with a haemolysing solution before being analysed using a Biosen C‐line Glucose and Lactate Analyzer (Biosen, EKF, Stoke on Trent, UK). Lactate turn point was determined by two independent researchers through visual inspection of the BLA–workload curve as the workload beyond which BLA began to increase exponentially.

### 2.7 Force platform and cable extension potentiometer

Two force platforms (OR6‐7, AMTI, Watertown, MA, USA) embedded in parallel directly underneath the VGSS treadmill measured ground reaction forces at 2000 Hz. Signals from the force platform passed to a strain gauge amplifier (MSA‐6, AMTI) with the gain of the vertical force component set to 4000. The amplified signal was sampled by the same data acquisition hardware and custom program as the cable extension potentiometer (previously described). The force platforms were hardware zeroed prior to each test. Custom MATLAB code (MATLAB 2023b, MathWorks, Natick, MA, USA) filtered the raw force data (50 Hz low‐pass fourth‐order Butterworth) and cable extension potentiometer data (20 Hz low‐pass fourth‐order Butterworth). The force platform and suspended participant should ideally be perpendicular; however, due to slight variability in suspension position and foot placement during jump landings relative to the force plate, a component of vGRF (*F_z_*) along the longitudinal axis of the body can be measured in the anterior–posterior axis (*Fy*). To account for this, vGRF was recalculated as the resultant of the *F_z_* and *F_y_* forces in accordance with Pythagorean theorem.

The following outcomes were computed post‐filtering for each individual jump using custom MATLAB code: peak vGRF (single highest value), relative mean breaking force (RMBF) and duration, relative mean propulsive force (RMPF) and duration, contact and flight times and centre of mass displacement during landing (i.e., jump depth). The start/end of the contact phase for each jump was determined at the point at which vGRF was above/below 5 standard deviations of the mean background vGRF noise measured in the flight phase of the preceding jump. The transition between breaking and propulsive phases was determined as the minimum turning point of the cable extension potentiometer (i.e., minimum centre of mass displacement), which was also used as a measure of jump depth. Each outcome was averaged per set of exercises. Force data are expressed as a percentage of 1 *g* bodyweight. Jump variability was examined for each outcome by computing their standard deviation from ∼130 to 150 jumps each participant performed for a given stage.

### 2.8 Perceptual measures

Participants scored three perceptual scales following each jumping stage. These included rating of perceived exertion (RPE; Borg 6–20 scale), discomfort (Borg‐CR10) and body instability (Modified Copper–Harper Body Control Scale). The latter scale was originally developed to quantify the mental workload of operators during aircraft handling and was applied in this study in the context of the participants’ perception of movement control when performing jumping under reduced bodyweight loading within the VGSS, in the same manner as in previous human spaceflight exercise countermeasure research (Attias et al., 2017; Harper Jr & Cooper, 1986). Participants were familiarised with the scales prior to exercise.

### 2.9 Maximal oxygen uptake graded exercise test

A GXT to volitional exhaustion was performed upright on an electronically braked cycle ergometer (Velotron, SRAM, Chicago, IL, USA) to obtain ${\dot{V}}_{O_{2}\max}$ for the normalisation of the metabolic cost of Lunar jumping. Cycle ergometry was selected to measure ${\dot{V}}_{O_{2}\max}$ as it is unknown whether it is at all possible to attain ${\dot{V}}_{O_{2}\max}$ during an incremental jumping test in simulated Lunar gravity. The GXT started at an initial workload of 50 W and increased linearly by 0.42 W s^−1^ (25 W min^−1^) whilst participants maintained a cadence ≥70 revolutions per minute. A plateauing of the ${\dot{V}}_{O_{2}}$–workload relationship (<5 mL min^−1^ W^−1^) across the final 50 W of the GXT was used as the primary criterion to verify that ${\dot{V}}_{O_{2}\max}$ had been reached (Niemeyer et al., 2020). Secondary criteria proposed by Wagner and colleagues were used to verify ${\dot{V}}_{O_{2}\max}$ in the absence of a ${\dot{V}}_{O_{2}}$ plateau, including RER_max_ ≥1.13, ≥96% of age‐predicted maximal heart rate (APMHR) (210 − age) and ≥93% of APMHR (208 − (0.7 × age)) (Wagner et al., 2020). All participants met at least one ${\dot{V}}_{O_{2}\max}$ criterion. A 15‐breath moving average was applied to the data and the highest ${\dot{V}}_{O_{2}}$ value was used to define ${\dot{V}}_{O_{2}\max}$ (Robergs et al., 2010).

### 2.10 Statistical analyses

The SPSS Statistics (Version 29.0.1.0, IBM Corp, Armonk, NY, USA) was used for statistical analysis. Statistical significance was set a priori at *P* < 0.05. Individual‐level relationships between jump height and each outcome measurement were modelled using first‐ or second‐order polynomial regression. The median coefficient of determination (*R* ^2^) and *P*‐value of the individual‐level relationships are reported in the manuscript; all data can be found in Supplementary Material 1. At each jumping stage, linear regression was performed between BLA and ${\dot{V}}_{O_{2}\max}$, BLA and fractional utilisation of aerobic capacity (%${\dot{V}}_{O_{2}\max}$), %${\dot{V}}_{O_{2}\max}$ and ${\dot{V}}_{O_{2}\max}$, peak vGRF and contact time, jump depth and contact time, RMBF and breaking phase duration and RMPF and propulsive phase duration. This resulted in nine *R* ^2^ values, one for each jump height; the median *R* ^2^ is reported in the manuscript and the *R* ^2^ for each jump height is reported in Supplementary Material 2. Data are presented as means ± standard deviation unless otherwise specified.

## 3 RESULTS

All participants completed the study with no adverse events. The percentage of participants that completed each stage was as follows: 30 cm (79%; 15/19), 35 cm (89%; 17/19), 40 cm (100%; 19/19), 45 cm (100%; 19/19), 50 cm (100%; 19/19), 55 cm (100%; 19/19), 60 cm (84%, 16/19), 65 cm (63%; 12/19) and 70 cm (37%; 7/19). The mean jump height was ≤1 cm relative to the target height at each stage and with a small standard deviation (±1–3 cm). The mean individual standard deviation of jump heights and frequencies for each stage was in the order of 2–3 cm and approximately one jump per minute, respectively, demonstrating that participants were able to consistently jump at the target height with a stable jumping frequency (Supporting information, Table S1).

### 3.1 Cardiorespiratory

**FIGURE 2:**
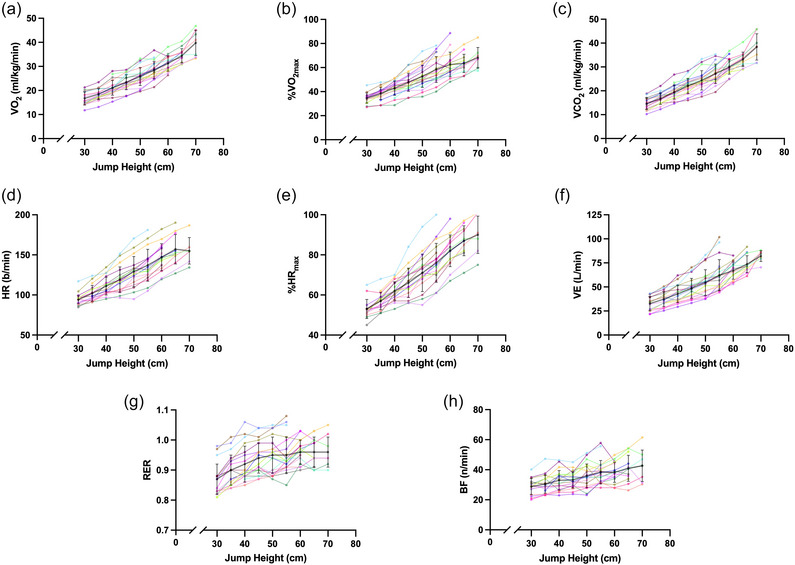
Cardiorespiratory responses to incremental jumping in simulated Lunar gravity. This figure displays individual responses (coloured lines) and group means ± SD (black line with error bars). BF, breathing frequency; HR, heart rate; RER, respiratory exchange ratio; ${\dot{V}}_{CO_{2}}$, volume of carbon dioxide production; ${\dot{V}}_{E}$, minute ventilation; ${\dot{V}}_{O_{2}}$, volume of oxygen consumption. [Correction made on 3rd September 2025, after first online publication: Figure 2 has been updated to reflect correct values of VO_2_ in panel A.]

Table 2 and Figure 2 display the cardiorespiratory responses to jumping in simulated Lunar gravity. Jump height displayed positive associations with all cardiorespiratory outcomes (median *R* ^2^ = 0.78–0.97). During the final jumping stage completed by each participant, oxygen uptake and heart rate reached their highest values of 71 ± 9% ${\dot{V}}_{O_{2}\max}$ (range: 59–88% ${\dot{V}}_{O_{2}\max}$) and 88 ± 9% HR_max_ (range: 75–101% HR_max_). Individuals with higher cardiorespiratory fitness levels (${\dot{V}}_{O_{2}\max}$) jumped at lower relative intensities; negative linear relationships (*R* ^2^ = 0.40–0.80) were identified between ${\dot{V}}_{O_{2}\max}$ and fractional utilisation of ${\dot{V}}_{O_{2}\max}$ (%${\dot{V}}_{O_{2}\max}$) at each jump height (Supporting information, Figure S3). Supporting information Figures S1 and S2 display group mean responses and box plots, respectively, for cardiorespiratory measures. Individual‐level relationships between jump height and each cardiorespiratory outcome can be found in Supporting information, Supplementary Material 1. Raw data can be found in Supplementary Material 3.

**TABLE 2:** Cardiorespiratory and metabolic responses to incremental jumping in simulated Lunar gravity.

	Target jump height (cm)										
Outcome	30	35	40	45	50	55	60	65	70	*R* ^2^	*P*
${\dot{V}}_{O_{2}}$ (%${\dot{V}}_{O_{2}\max}$)	35 ± 4	39 ± 5	43 ± 6	48 ± 8	53 ± 9	58 ± 11	62 ± 11	63 ± 8	68 ± 9	0.96	<0.0001
${\dot{V}}_{O_{2}}$ (ml kg^−1^ min^−1^)	17 ± 3	18 ± 2	21 ± 3	23 ± 3	26 ± 4	28 ± 4	31 ± 3	34 ± 3	40 ± 6	0.96	<0.0001
${\dot{V}}_{CO_{2}}$ (ml kg^−1^ min^−1^)	15 ± 3	17 ± 2	19 ± 3	22 ± 3	24 ± 4	27 ± 4	30 ± 3	33 ± 3	38 ± 6	0.96	<0.0001
HR (%HR_max_)	53 ± 5	57 ± 4	62 ± 5	66 ± 8	71 ± 10	76 ± 10	82 ± 8	87 ± 8	90 ± 10	0.97	<0.0001
HR (beats min^−1^)	95 ± 8	102 ± 10	111 ± 12	119 ± 16	129 ± 20	137 ± 21	147 ± 18	157 ± 19	155 ± 18	0.97	<0.0001
${\dot{V}}_{E}$ (L min^−1^)	33 ± 8	37 ± 8	43 ± 9	49 ± 11	54 ± 14	62 ± 17	67 ± 9	74 ± 9	82 ± 6	0.94	0.0002
RER	0.87 ± 0.06	0.90 ± 0.05	0.92 ± 0.06	0.94 ± 0.06	0.95 ± 0.06	0.95 ± 0.06	0.96 ± 0.04	0.96 ± 0.05	0.96 ± 0.05	0.84	0.0040
BF (n min^−1^)	29 ± 6	31 ± 6	33 ± 7	33 ± 6	36 ± 8	38 ± 8	38 ± 7	41 ± 9	43 ± 11	0.78	0.0167
Blood lactate (mmol L^−1^)^b^	1.7 ± 0.4	1.9 ± 0.8	2.3 ± 1.0	2.6 ± 1.3	3.4 ± 1.6	4.1 ± 1.9	4.2 ± 1.2	5.2 ± 1.6	5.5 ± 1.7	0.98^a^	0.0004

*Note*: Sample sizes are as follows: 30 cm (*n* = 15), 35 cm (*n* = 17), 40 cm (*n* = 19), 45 cm (*n* = 19), 50 cm (*n* = 19), 55 cm (*n* = 19), 60 cm (*n* = 16), 65 cm (*n* = 12) and 70 cm (*n* = 7). Due to signal loss, sample sizes for heart rate data are as follows: 30 cm (*n* = 14), 35 cm (*n* = 16), 40 cm (*n* = 17), 45 cm (*n* = 17), 50 cm (*n* = 16), 55 cm (*n* = 17), 60 cm (*n* = 15), 65 cm (*n* = 10) and 70 cm (*n* = 6). The sample size for blood lactate at 50 cm is *n* = 18 due to a missing sample. *R* ^2^ and *P*‐values reflect the median individual *R* ^2^ and *P*‐value for the relationship between jump height and the given measurement. All cardiorespiratory measures were modelled using linear regression. ^a^Blood lactate was modelled against jump height using 2nd order polynomial (quadratic) regression. ^b^Resting blood lactate = 1.6 ± 0.4 mmol L^−1^ (*n* = 19). Abbreviations: BF, breathing frequency; HR, heart rate; RER, respiratory exchange ratio; ${\dot{V}}_{CO_{2}}$, volume of carbon dioxide production; ${\dot{V}}_{E}$, minute ventilation; ${\dot{V}}_{O_{2}}$, volume of oxygen consumption; ${\dot{V}}_{O_{2}\max}$, maximal oxygen uptake.

### 3.2 Blood lactate

**FIGURE 3:**
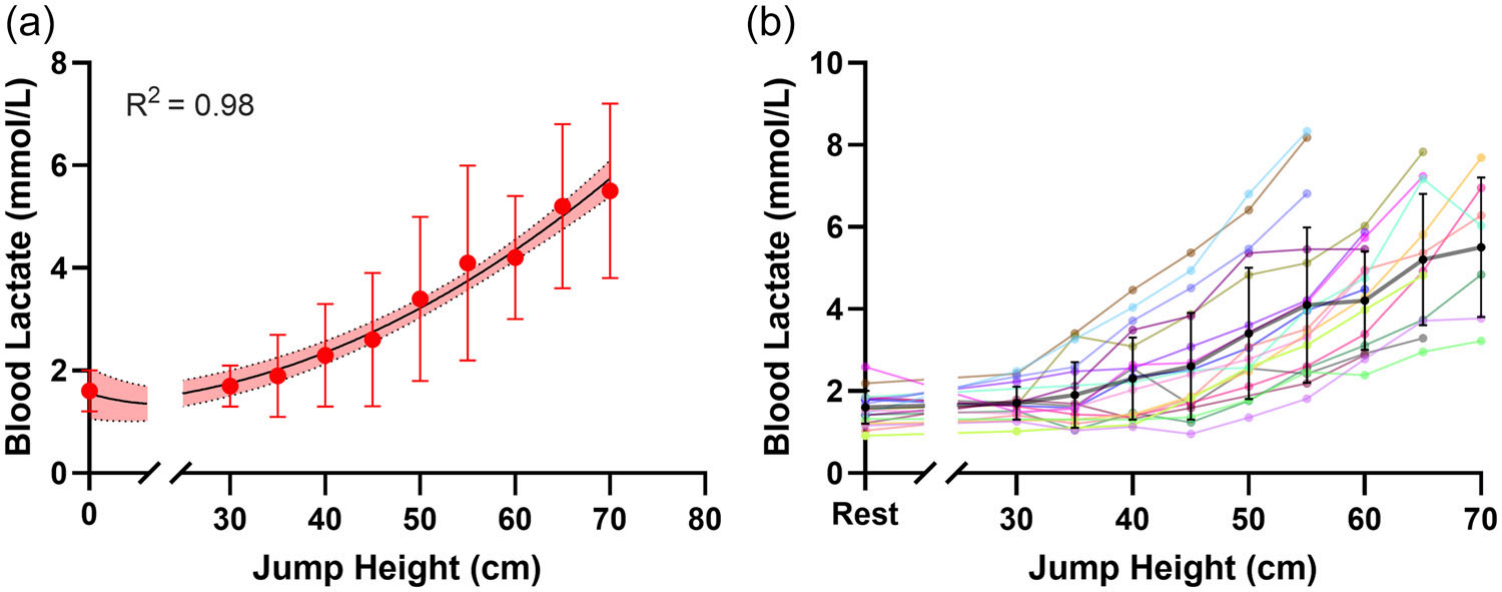
Blood lactate concentrations during incremental jumping in simulated Lunar gravity. *R* ^2^ is for a quadratic regression model. (a) Cohort means (± SD) and a quadratic curve line shaded in red with 95% confidence intervals. (b) Individual responses (coloured lines) and group means (± SD) as black line and error bars.

Blood lactate concentration increased in a curvilinear manner with jump height from (mean ± SD) resting values of 1.6 ± 0.4 mmol L^−1^ up to 5.5 ± 1.7 mmol L^−1^ (quadratic *R* ^2^ = 0.98, *P* = 0.0004) (Table 2). Figure 3 displays the cohort and individual blood lactate responses at each jump height. Supporting information Figure S4 displays box plots for BLA at each jumping stage. During the final jumping stage for each participant, BLA was on average 5.8 ± 1.7 mmol L^−1^ and ranged between ∼3 and 8 mmol L^−1^. At the 55 cm jump height stage (the last stage completed by all 19 participants), BLA responses were highly variable between individuals, ranging from ∼2 to 8 mmol L^−1^. The point at which blood lactate began to rise exponentially occurred, on average, at jump heights of 45 ± 8 cm, 48 ± 10% ${\dot{V}}_{O_{2}\max}$ and 67 ± 11% HR_max_. The correlation between cardiovascular fitness (${\dot{V}}_{O_{2}\max}$) and BLA at each stage was weak (*R* ^2^ = 0.12–0.23) (Supporting information, Figure S5). Positive linear relationships of varying strengths were identified between fractional utilisation of ${\dot{V}}_{O_{2}\max}$ (%${\dot{V}}_{O_{2}\max}$) and BLA at each jumping stage (*R* ^2^ = 0.10–0.61) (Supporting information, Figure S6). Individual‐level relationships between jump height and BLA can be found in Supporting information, Supplementary Material 1. Raw data can be found in Supplementary Material 3.

### 3.3 Biomechanics

**FIGURE 4:**
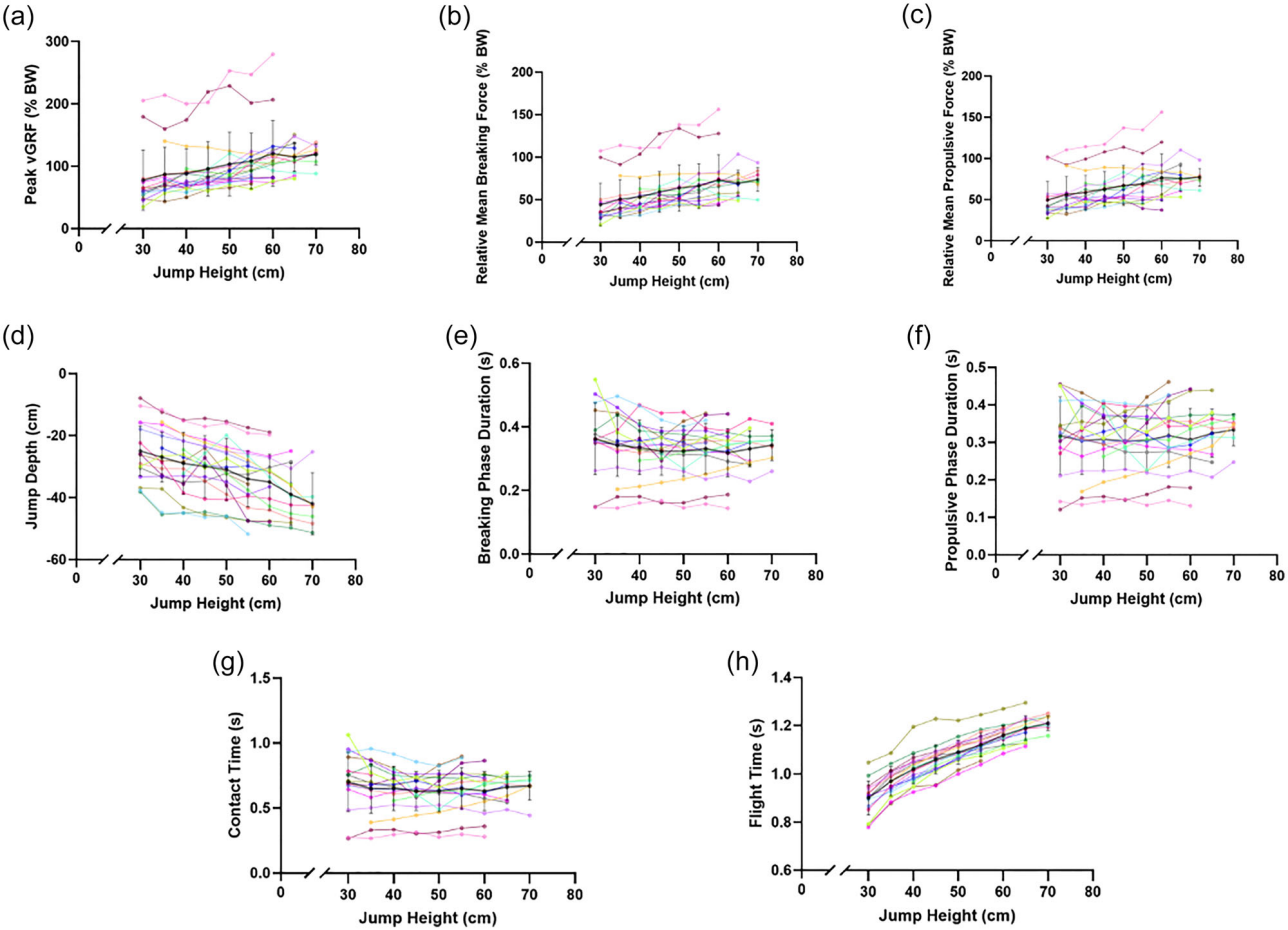
Biomechanical outcomes for jumping in simulated Lunar gravity. The figure displays individual responses (coloured lines) and group means ± SD (black line with error bars). BW, bodyweight; vGRF, vertical ground reaction force.

Table 3 and Figure 4 display the biomechanical outcomes during incremental jumping in simulated Lunar gravity. Linear relationships were observed between jump height and jump depth (*R* ^2^ = 0.82), jump frequency (*R* ^2^ = 0.81), flight time (*R* ^2^ = 0.96), peak vGRF (*R* ^2^ = 0.77), RMBF (*R* ^2^ = 0.80) and RMPF (*R* ^2^ = 0.72). No relationships were observed between jump height and contact time (*R* ^2^ = 0.24), breaking phase duration (*R* ^2^ = 0.30) and propulsive phase duration (*R* ^2^ = 0.12). Supporting information Figures S7 and S8 display group mean responses and box plots, respectively, for biomechanical outcomes. Individual‐level relationships between jump height and each biomechanical measure can be found in Supporting information, Supplementary Material 1. Individual‐level standard deviations for each biomechanical outcome can be found in Supporting information, Table S1. At each jump height, negative linear relationships were observed between peak vGRF and contact time (median *R* ^2^ = 0.74), jump depth and contact time (median *R* ^2^ = 0.68), RMBF and breaking phase duration (median *R* ^2^ = 0.76) and RMPF and propulsive phase duration (median *R* ^2^ = 0.79) (Supporting information, Supplementary Material 2 and Figures S11–S14). Raw data can be found in Supplementary Material 4.

**TABLE 3:** Biomechanical outcomes for incremental jumping in simulated Lunar gravity.

Outcome	Target Jump Height (cm)									*R* ^2^	*P*
	30	35	40	45	50	55	60	65	70		
Jump height (cm)	30 ± 1	36 ± 1	41 ± 1	46 ± 2	51 ± 1	55 ± 2	60 ± 2	66 ± 3	71 ± 3	0.99	<0.0001
Jump depth (cm)	−25 ± 10	−27 ± 10	−29 ± 9	−30 ± 9	−31 ± 10	−34 ± 10	−35 ± 9	−38 ± 9	−43 ± 7	0.82	0.0017
Jump frequency (jumps/min)	38 ± 5	37 ± 5	36 ± 4	36 ± 3	35 ± 3	34 ± 3	34 ± 3	32 ± 2	32 ± 2	0.81	0.0018
Contact time (s)	0.68 ± 0.21	0.65 ± 0.19	0.64 ± 0.17	0.63 ± 15	0.63 ± 0.16	0.65 ± 0.16	0.63 ± 0.16	0.66 ± 0.11	0.68 ± 0.08	0.24	0.1788
Flight time (s)	0.91 ± 0.08	0.98 ± 0.06	1.03 ± 0.06	1.06 ± 0.06	1.10 ± 0.05	1.13 ± 0.05	1.17 ± 0.05	1.19 ± 0.05	1.22 ± 0.03	0.96	<0.0001
Breaking phase duration (s)	0.36 ± 0.11	0.34 ± 0.10	0.34 ± 0.09	0.32 ± 0.08	0.32 ± 0.08	0.33 ± 0.08	0.32 ± 0.08	0.33 ± 0.06	0.34 ± 0.05	0.30	0.1780
Propulsive phase duration (s)	0.32 ± 0.10	0.30 ± 0.09	0.31 ± 0.08	0.30 ± 0.08	0.31 ± 0.08	0.32 ± 0.09	0.31 ± 0.08	0.32 ± 0.06	0.33 ± 0.04	0.12	0.4190
Peak vGRF (% bodyweight)	78 ± 48	87 ± 44	89 ± 40	96 ± 44	104 ± 51	108 ± 51	120 ± 53	115 ± 23	119 ± 17	0.77	0.0047
Mean breaking force (% bodyweight)	44 ± 25	51 ± 23	53 ± 22	59 ± 24	64 ± 27	66 ± 26	73 ± 30	69 ± 15	74 ± 14	0.80	0.0050
Mean propulsive force (% bodyweight)	50 ± 22	56 ± 22	58 ± 21	63 ± 21	67 ± 24	69 ± 24	77 ± 29	76 ± 15	77 ± 11	0.72	0.0085

*Note*: Sample sizes are as follows: 30 cm (*n* = 15), 35 cm (*n* = 17), 40 cm (*n* = 19), 45 cm (*n* = 19), 50 cm (*n* = 19), 55 cm (*n* = 19), 60 cm (*n* = 16), 65 cm (*n* = 12) and 70 cm (*n* = 7). Jump depth reflects negative centre of mass displacement during landing (standing = 0 cm). *R* ^2^ and *P*‐values reflect the median individual *R* ^2^ and *P*‐value for the relationship between jump height and given measurement; all were modelled using linear regression. Abbreviations: vGRF, vertical ground reaction force.

### 3.4 Perceptual

**FIGURE 5:**
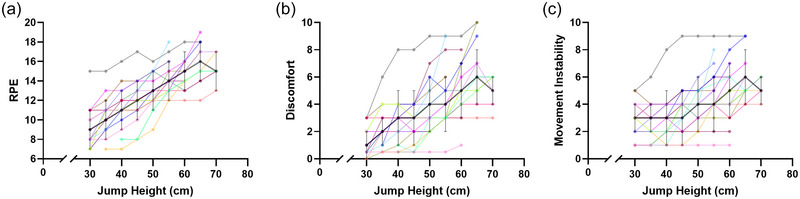
Perceptual responses to incremental jumping in simulated Lunar gravity. The figure displays individual responses (coloured lines) and group means ± SD (black line with error bars). RPE, rating of perceived exertion.

The RPE, discomfort and movement instability for each jumping stage are displayed in Table 4 and Figure 5. All measures were strongly linearly associated with jump height (*R* ^2^ = 0.74–0.93); individual‐level relationships can be found in Supporting information, Supplementary Material 1. Supporting information Figures S8 and S9 display the group mean responses and box plots, respectively, for perceptual measures at each jumping stage. Raw data can be found in Supplementary Material 3.

**TABLE 4:** Perceptual responses to incremental jumping in simulated Lunar gravity.

Outcome	Target Jump Height (cm)									*R* ^2^	*P*
	30	35	40	45	50	55	60	65	70		
RPE	9 ± 2	10 ± 2	11 ± 2	12 ± 2	13 ± 2	14 ± 2	15 ± 2	16 ± 2	15 ± 2	0.93	0.0004
Discomfort	1 ± 1	2 ± 1	3 ± 2	3 ± 2	4 ± 2	4 ± 2	5 ± 2	6 ± 2	5 ± 1	0.82	0.0039
Movement instability	3 ± 1	3 ± 1	3 ± 2	3 ± 2	4 ± 2	5 ± 2	5 ± 2	6 ± 2	5 ± 1	0.74	0.0074

*Note*: *n* = 15 at 60 cm due to missing perceptual data. *R* ^2^ and *P*‐values reflect the median individual *R* ^2^ and *P*‐value for the relationship between jump height and given measurement; all were modelled using linear regression. Abbreviations: RPE, rating of perceived exertion.

## 4 DISCUSSION

### 4.1 Summary of key findings

The present study examined the acute physiological responses to bodyweight jumping at incremental heights performed at ∼20% bodyweight, simulating Lunar gravity, to provide new insight into the potential use of jumping as a form of exercise in surface habitats on the Moon. Cardiorespiratory measures (HR, ${\dot{V}}_{O_{2}}$, ${\dot{V}}_{CO_{2}}$, ${\dot{V}}_{E}$ and BF) increased linearly with jump height (*R* ^2^ = 0.78–0.97) and participants achieved, on average, exercise intensities of 70 ± 9% ${\dot{V}}_{O_{2}\max}$ and 88 ± 9% HR_max_ during their final jumping stage; however, with large inter‐individual variability. Blood lactate concentrations increased in a curvilinear manner (*R* ^2^ = 0.98) up to 5.8 ± 1.7 mmol L^−1^. Peak vGRFs during jump landings/take‐offs were linearly related to jump height (*R* ^2^ = 0.77) and averaged ∼80–120% bodyweight during the jumping protocol, but in some cases were up to ∼200–300% bodyweight. Relative mean breaking and propulsive forces were similar in magnitude, ranging from ∼40% to 80% bodyweight and increased linearly with jump height (*R* ^2^ = 0.72–0.80). Flight time increased linearly with jump height (*R* ^2^ = 0.96) whilst contact time displayed no relationship (*R* ^2^ = 0.24). The constituent parts of contact time – breaking and propulsive phases – also displayed no relationship with jump height (*R* ^2^ = 0.12–0.30). Jump depth was negatively associated with jump height (*R* ^2^ = 0.82). RPE, discomfort and body instability increased linearly with jump height (*R* ^2^ = 0.74–0.94). These findings demonstrate that continuous bodyweight jumping in simulated Lunar gravity can allow for exercise across a range of submaximal cardiovascular intensities, with and without blood lactate accumulation and can induce vGRFs equivalent to standing, walking and running on Earth.

### 4.2 Implications of findings to planetary exploration missions

Identifying new ways of exercising using less hardware has been an area of contemporary importance for space agencies planning surface exploration‐class missions to the Moon and Mars. The present study demonstrated that bodyweight jumping in simulated Lunar gravity can elicit a range of submaximal exercise intensities (cohort means: ∼35–70% ${\dot{V}}_{O_{2}\max}$ and ∼50–90% HR_max_) as a function of jump height. Blood lactate concentrations also progressively rose in a curvilinear manner with respect to jump height (*R* ^2^ = 0.98) up to a mean of ∼5–6 mmol L^−1^ during the final jumping stage. Large inter‐individual variability was evident and significant negative associations were identified between cardiorespiratory fitness (${\dot{V}}_{O_{2}\max}$) and the fractional utilisation of ${\dot{V}}_{O_{2}\max}$ (%${\dot{V}}_{O_{2}\max}$) at each jump height (*R* ^2^ = 0.40–0.80). For reference, during the final jumping stage completed by each participant, the cardiorespiratory and metabolic responses ranged between ∼60% and 90% ${\dot{V}}_{O_{2}\max}$ and ∼75% and 100% HR_max_ with blood lactate concentrations ranging from ∼3 to 8 mmol L^−1^.

Exercise intensity plays a key role in mediating physiological adaptations to exercise (MacInnis & Gibala, 2017). In endurance training programmes, the distribution of training volumes (e.g., cycling/running distance) to specific intensity ‘zones’ or ‘domains’ is recognized to have an important role in optimizing physiological adaptations to endurance exercise and athletic performance (Rosenblat et al., 2019; Seiler, 2012; Stöggl & Sperlich, 2014). Pyramidal and polarised training intensity distribution models are commonly employed by endurance athletes (Stöggl & Sperlich, 2015). In these models, a large percentage of training volume is devoted to the ‘moderate‐intensity’ domain (∼70–80%) at workloads that do not result in blood lactate accumulation, with lesser training volumes (∼0–20%) devoted to training in the ‘heavy‐intensity’ domain in which blood lactate accumulation manifests but can be sustained at elevated levels (e.g., ‘threshold’ or ‘tempo’ training) and/or ‘severe‐intensity’ domain in which blood lactate progressively accumulates (e.g., high‐intensity interval training) (Seiler, 2012).

Astronauts perform cardiovascular exercise onboard the International Space Station at workloads equivalent to 70–100% of their pre‐flight ${\dot{V}}_{O_{2}\max}$ using a combination of moderate‐intensity continuous training and higher intensity interval training (English et al., 2020). Our findings suggest that jumping in Lunar gravity could be used to perform exercise in the moderate‐ and heavy‐intensity domain, as evidenced by the occurrence of exponential rises in blood lactate concentrations (‘lactate turn point’; LT). Interestingly, the exercise intensity at which LT manifested during jumping was far lower (∼50% ${\dot{V}}_{O_{2}\max}$ and ∼70% HR_max_) than those reported in adults during incremental upright cycling and running exercise in 1 *g* (∼65–85% ${\dot{V}}_{O_{2}\max}$ and 80–90% HR_max_) (Gordon et al., 2017; Messonnier et al., 2013). This may be a result of the jumping being performed in an almost horizontal position (i.e., 9.5° head‐up tilt) and normalising the cardiorespiratory responses to those of the ${\dot{V}}_{O_{2}\max}$ test which was performed using upright cycle ergometry in 1 *g*. Both body position and exercise modality can influence ${\dot{V}}_{O_{2}\max}$, leading to a plausible scenario in which the fractional utilisation of ${\dot{V}}_{O_{2}\max}$ during jumping could have been underestimated as ${\dot{V}}_{O_{2}\max}$ and HR_max_ in a supine position can be ∼10–20% lower relative to when performed upright (Dillon et al., 2021). Dillon et al. suggested several mechanisms by which ${\dot{V}}_{O_{2}\max}$ may be reduced in the supine relative to upright cycling posture including: (1) attenuated preload due to elevated intrathoracic pressure from cephalic displacement of the diaphragm and abdominal organs, (2) attenuated sympathetic activation (decreasing HR and stroke volume via reduced venoconstriction), and (3) attenuated skeletal muscle blood flow via a reduction in muscle perfusion pressure due to a diminished hydrostatic pressure gradient from gravity (Dillon et al., 2021). In addition, previous research has observed that the manifestation of lactate turn point can occur at lower rates of oxygen consumption (∼7%) and heart rates (∼11%) in a supine relative to upright cycling position (Wehrle et al., 2021).

The cardiovascular benefits of exercising close to ${\dot{V}}_{O_{2}\max}$ (e.g., interval training at ≥90% ${\dot{V}}_{O_{2}\max}$) are well recognised (Maturana et al., 2021; Rosenblat et al., 2019). The present results suggest that jumping at ∼20% bodyweight may not be able to elicit exercise intensities ≥90% ${\dot{V}}_{O_{2}\max}$ for most individuals, particularly as it was found that fitter individuals jumped at lower relative intensities for a given jump height. However, we contend this remains in question. First, participants were asked if they could complete the next jumping stage in the preceding rest period, and if not, the test was then terminated. Should the participant have attempted the next stage, it is possible that higher exercise intensities could have been reached. In addition, if the participant was operating in the ‘severe’ exercise domain, characterised by inability to maintain physiological homeostasis, then one would expect that they could eventually attain ${\dot{V}}_{O_{2}\max}$ if the stage duration was extended (Burnley & Jones, 2007). Second, the final jumping stage was limited to 70 cm due to headroom restrictions of the hypogravity analogue and participants able to complete this stage would have likely been able to attempt further stages at higher jump heights and therefore higher exercise intensities. Third, some participants had to terminate the jumping test prematurely due to an inability to control side‐to‐side swinging caused by the body being suspended as a pendulum. Fourth, as previously mentioned, the orientation of the body during jumping relative to the ${\dot{V}}_{O_{2}\max}$ test (upright cycle ergometry) may have led to underestimations in the relative exercise intensity. Further research is warranted to establish whether it is possible to jump within the ‘severe‐intensity’ exercise domain in Lunar gravity by employing protocols commensurate with the identification of demarcations of the heavy–severe transition such as critical power, respiratory compensation point and/or maximal lactate steady‐state and also whether it is possible to attain ${\dot{V}}_{O_{2}\max}$ (Gaskill et al., 2001; Goodwin et al., 2007). The question as to whether jumping in Lunar gravity may be limited by a ‘metabolic ceiling’, however, cannot be entirely dismissed. Given the negative linear relationship between fractional utilisation of ${\dot{V}}_{O_{2}\max}$ (%${\dot{V}}_{O_{2}\max}$) and aerobic fitness (${\dot{V}}_{O_{2}\max}$) at each jumping stage (Supporting information, Figure S3), it remains plausible that, particularly in individuals with higher levels of cardiovascular fitness, jumping as high as possible in Lunar gravity might only elicit submaximal intensities that could limit the potential effectiveness of this equipment‐free training method. This scenario thus prompts one to consider whether jumping on the Moon may need to be supplemented by additional equipment (e.g., a subject‐loading device) or complementary techniques (e.g., blood flow restriction) to augment the physiological demands (Hughes et al., 2022). It is valuable to add for context that the average ${\dot{V}}_{O_{2}\max}$ of participants in the present study was 50 mL kg^−1^ min^−1^, which is somewhat higher than the ${\dot{V}}_{O_{2}\max}$ of International Space Station astronauts pre‐flight (∼40 mL kg^−1^ min^−1^) (Scott et al., 2023).

The ground reaction force data have implications for both musculoskeletal loading and the metabolic demands of jumping in hypogravity. In accordance with the impulse–momentum theory, the net vertical force (impulse or change in momentum) required to jump a specific height for a given body mass is constant, with an inverse relationship existing between the sum of external forces acting on the body and the time interval over which they are applied. Flight time increased linearly with jump height, but contact time and its constituent phases – breaking and propulsive phase durations – showed no relationship with jump height, corroborating previous findings by Weber et al. that observed contact time remained stable when hopping at variable heights in simulated hypogravity (0.16 *g*, 0.27 *g*, 0.38 *g* and 0.70 *g*), (Weber et al., 2019). This would imply that it is the magnitude of force that is being selectively manipulated to increase jump height, as opposed to the time interval over which force is applied, given that participants had the freedom to self‐select jumping frequency. Peak vGRF and relative mean breaking and propulsive forces scaled linearly with jump height and are again comparable to previous findings examining hopping in hypogravity (Weber et al., 2019). Inter‐individual variability was evident for the vGRF measures that can be attributed to the jumping strategy employed by the participant, with ‘stiffer’ jumps resulting in shorter contact phases and higher vGRFs and vice versa (Price et al., 2024). Indeed, there were strong negative linear relationships between contact time and peak vGRF at each jump height and the relative mean breaking and propulsive forces were negatively associated with their respective durations at each jump height (see Supporting information, Figures S11–S14). For instance, the average peak vGRF was ∼80–120% bodyweight across jump heights, but two participants were achieving ∼200–300% bodyweight and had the shortest contact times of ∼250–350 ms versus the cohort average of ∼600–700 ms. For reference, vGRF values during walking and running on Earth are reported to be ∼120 ± 10% and ∼240 ± 20% bodyweight, respectively (Cavanagh et al., 2010). Jump depth, an indication of hip, knee and/or ankle flexion, increased linearly with jump height but was negatively associated with contact time at any given jump height. This may have implications for muscular adaptations, as, for instance, hypertrophy appears to be influenced by the exercise range of motion and muscle length at which the exercise is performed (Kassiano et al., 2023; Schoenfeld & Grgic, 2020). Jumping strategy (e.g., stiffness and depth) can be manipulated by the individual and, therefore, can be used as a method to modify musculoskeletal loading (Price et al., 2024). In accordance with the bone mechanostat hypothesis, jumping on the Moon could impose frequent ground impact forces up to several times bodyweight and shear stresses on bone via muscle forces, both of which appear to be important mechanical stimuli for bone remodelling (Frost, 2003; Kohrt et al., 2009). Furthermore, jumping on the Moon requires generation of muscular forces during concentric–eccentric muscle contractions that may also benefit musculoskeletal health due to the recognized benefits of impact loading and the stretch–shortening cycle (SSC) as a disuse/spaceflight countermeasure, as well as in general and athletic populations (Gruber et al., 2019; Kramer, Gollhofer et al., 2017; Oxfeldt et al., 2019; Stojanović et al., 2017).

Plyometric exercises typically aim for contact times <250 ms to utilise the so‐called ‘fast’ SSC, as longer contact times (e.g., >250 ms) are found to reduce power output due to a diminished utilisation of the SSC (Flanagan & Comyns, 2008; Wilson et al., 1991). Jumping in simulated Lunar gravity was found to result in average contact times of ∼600–700 ms across jump heights, but two participants jumped with contact times of ∼250–350 ms. It is entirely possible that contact times <250 ms are possible if instructed to jump with a ‘stiff‐legged’ take‐off/landing strategy. Different jumping strategies (e.g., ‘stiff’ vs. ‘compliant’) would likely modify musculoskeletal loading and the metabolic demand of jumping, which has implications for jump training programming. Use of a ‘stiff‐legged’ jumping strategy with short contact times (e.g., <250 ms) would result in high muscle power outputs and impact forces onto weight‐bearing skeletal regions (e.g., tibia, femoral neck and lumbar spine) that could increase bone mineral density, tendon stiffness and neuromuscular performance. In contrast, a compliant jumping strategy with longer contact times (e.g., >250 ms) would result in reduced utilisation of the SSC that, in theory, would evoke a greater metabolic response that could improve local muscle oxidative capacity and cardiovascular fitness. The long‐term effects of jumping in hypogravity remain to be studied, but it seems plausible that a well‐constructed jump training programme could elicit positive adaptations within the musculoskeletal and cardiovascular systems and improve physical performance, on the Moon or Mars (Kramer et al., 2019; Markovic & Mikulic, 2010; Oxfeldt et al., 2019; Ramírez‐Campillo et al., 2014; Ramírez‐delaCruz et al., 2022; Teoh, 2018).

Ratings of perceived exertion and discomfort increased linearly with jump height and are consistent with previous research examining the perceptual responses to incremental exercise (Muscat et al., 2015). Movement instability also increased linearly with jump height up to a mean value of 6/10 (‘tolerable deficiencies’), but with large variability between participants. Participants were suspended on their back via a pelvic to thorax/head support plate, in which it is impossible to fall forward/back. Due to being suspended as a pendulum, however, side‐to‐side swinging developed in some cases and could progressively worsen unless the participant could restabilise the jumping path. This is certainly unique to horizontal/head‐up tilt bodyweight suspension analogues; however, restrictions to and exacerbations of, certain movements must be considered with respect to movement in a real hypogravity environment. It seems prudent for this reason to examine how difficult it is to jump continuously at various heights in simulated hypogravity during vertical bodyweight suspension, as this system can allow for degrees‐of‐freedom in all three planes of motion that will impose a more realistic challenge to body control when jumping.

The present study explored jumping in simulated Lunar gravity (0.17 *g*). Missions to Mars, however, will expose astronauts to 0.38 *g*. It is logical to assume that bodyweight jumping on Mars, relative to the Moon, will elicit higher exercise intensities at a given jump height. It is plausible that jumping could elicit metabolic rates in the range of ∼70–100% ${\dot{V}}_{O_{2}\max}$ more feasibly in Martian gravity. Incremental jumping in hypogravity may also have potential value as a method to evaluate astronaut fitness in‐mission on the Moon/Mars, particularly as the present study observed all participants exhibiting an exponential rise in BLA concentration with respect to jump height, the workload at which this begins to manifest being a well‐recognised performance indicator for endurance‐related tasks (Heuberger et al., 2018). Further research is necessary to establish the probability of falls and/or identification of adverse biomechanics during jumping in hypogravity to identify any potential risks of jumping in hypogravity. There is also scope to investigate the acute physiological responses to various other forms of jumping (e.g., alternating single‐legged jumping, lunge jumping and squat jumping).

### 4.3 Limitations

An intrinsic limitation of using head‐up tilted bodyweight suspension is the use of ropes to suspend each body segment. During dynamic movements where the body's centre of mass displaces along the body's longitudinal axis, which is oriented perpendicular to the suspension ropes, an angular displacement of the ropes will occur. The suspension ropes, therefore, act in a pendulum‐like manner, where Earth's gravity will lead to an acceleration of the rope (and the suspended body) towards a vertical orientation. This acceleration will lead to an additional acceleration acting on the suspended body, in addition to the target gravitational acceleration acting along the body's longitudinal axis. For a given vertical impulse, this pendulum effect would have reduced jump height and flight time, thus increasing jumping frequency (jumps per minute), relative to without the pendulum (i.e., if jumping on the actual Lunar surface). The ground reaction forces experienced by the jumper are unaffected by pendulum swinging as no energy is added to or lost from the system (assuming trivial air resistance and friction); however, it is logical to assume that performing the same amount of work per jump at an increased frequency would amplify the cardiorespiratory and metabolic responses to jumping at a given height.

Due to challenges faced by participants in maintaining correct posture during jumping in the hypogravity analogue, in terms of their anteroposterior orientation, the ropes suspending the pelvis and thorax/head segments were locked in place, thus preventing anteroposterior displacement of the body. Further research is needed to determine whether the challenges that participants faced in maintaining correct posture with the suspension ropes not locked in place (i.e., allowing for anteroposterior movement) could be overcome with sufficient training. Understanding whether it is possible to jump continuously at high heights in reduced gravity settings would provide valuable information as to the potential use of jumping as a planetary countermeasure. A high probability of falling over, movement instability and/or adverse biomechanics could impose unnecessary risks to astronaut health (e.g., falls‐ or strain‐related injury) and would likely influence all the measured responses to jumping if the user must continually stop and restart jumping and/or require far greater levels of postural stability and movement co‐ordination compared to when performed during head‐up tilt bodyweight suspension.

The influence of the body's orientation must also be considered with respect to gravity, for this can have a significant impact on cardiovascular function (Whittle et al., 2022). It has been shown that ${\dot{V}}_{O_{2}\max}$ and HR_max_ can be ∼20% and ∼10% lower, respectively, when performed in a supine relative to an upright position during cycle ergometry (Dillon et al., 2021). This is important to consider as participants in the present study performed a GXT in an upright posture on a cycle ergometer, whilst the jumping exercise was performed in a 9.5° head‐up tilt from the horizontal axis. If the graded test was performed at a 9.5° head‐up tilt position, it is possible that participants may have been exercising much closer to their ${\dot{V}}_{O_{2}\max}$ and HR_max_ attainable in this position. Furthermore, the exercise modality used can also influence ${\dot{V}}_{O_{2}\max}$; for instance, ${\dot{V}}_{O_{2}\max}$ during cycling can be ∼5–10% lower relative to treadmill running (Zwiren et al., 1991). As it was unclear whether ${\dot{V}}_{O_{2}\max}$ could be attained during an incremental jumping test at ∼20% bodyweight, the present study opted to measure ${\dot{V}}_{O_{2}\max}$ using cycle ergometry. Readers must, therefore, remain aware of the differences in posture and exercise mode when interpreting %${\dot{V}}_{O_{2}\max}$ and %HR_max_ outcomes that are expressed relative to maximum values attained during the GXT performed on an upright cycle ergometer.

Missions to Mars will expose astronauts to 0.38 *g*, and therefore it would be valuable to characterise the acute physiological responses to incremental jumping in simulated Martian gravity. Given that bodyweight on Mars will be approximately double that on the Moon, it is logical to assume that the exercise intensity will be greater for a given jump height, which may allow for exercise intensities in the range of 70–100% ${\dot{V}}_{O_{2}\max}$ to be more easily achievable. There is also plenty of scope to investigate various other forms of jumping in hypogravity that may impose a greater metabolic cost when jumping at a given height, which may be favourable to eliciting near‐maximal/maximal exercise intensities (e.g., alternating single‐legged jumping, lunge jumping and squat jumping).

### 4.4 Conclusions

Bodyweight jumping in simulated Lunar gravity at incremental heights resulted in linear increases in cardiorespiratory demand (e.g., heart rate and oxygen consumption) up to 71 ± 9% ${\dot{V}}_{O_{2}\max}$ and 88 ± 9% HR_max_ and a curvilinear increase in blood lactate concentrations up to 5.8 ± 1.7 mmol L^−1^. Inter‐individual variability was notable and participants with higher levels of cardiovascular fitness required a lower fractional utilization of ${\dot{V}}_{O_{2}\max}$ at a given jump height. The cardiorespiratory and metabolic demand observed during the final jumping stage completed by each participant ranged from ∼60% to 90% ${\dot{V}}_{O_{2}\max}$ and ∼75 to 100% HR_max_ with blood lactate concentrations ranging from ∼3 to 8 mmol L^−1^. Peak vGRFs were, on average, ∼80–120% bodyweight, but in some cases were up to ∼200–300% bodyweight, due to use of a ‘stiffer’ jump landing/take‐off technique. In summary, bodyweight jumping in simulated Lunar gravity can permit exercise in the moderate‐ and heavy‐intensity exercise domains with frequent impact forces that may induce musculoskeletal and cardiovascular adaptation and/or protection against disuse‐induced deconditioning.

## AUTHOR CONTRIBUTIONS

The study was conducted in the Aerospace Medicine & Rehabilitation Laboratory (Northumbria University, UK). All authors contributed to (1) the conception or design of the work, (2) the acquisition, analysis or interpretation of data for the work and (3) drafting the work or revising it critically for important intellectual content. All authors have read and approved the final version of this manuscript and agree to be accountable for all aspects of the work in ensuring that questions related to the accuracy or integrity of any part of the work are appropriately investigated and resolved. All persons designated as authors qualify for authorship, and all those who qualify for authorship are listed.

## CONFLICT OF INTEREST

None decalred.

## FUNDING INFORMATION

None.

## Supporting information



Supplementary Figures S1–S14.

Supplementary Material 1

Supplementary Material 2

Supplementary Material 3

Supplementary Material 4

Supplementary Table S1. Biomechanical outcomes for incremental jumping in simulated Lunar gravity.

Supplementary Video S1.

## Data Availability

Data pertaining to this study can be found in the Supporting information.

## References

[eph13866-bib-0001] Ade, C. , Broxterman, R. , Craig, J. , Schlup, S. , Wilcox, S. , & Barstow, T. (2014). Relationship between simulated extravehicular activity tasks and measurements of physical performance. Respiratory Physiology & Neurobiology, 203, 19–27. 25169116 10.1016/j.resp.2014.08.007

[eph13866-bib-0002] Ade, C. J. , Broxterman, R. M. , Craig, J. C. , Schlup, S. J. , Wilcox, S. L. , & Barstow, T. J. (2015). Standardized exercise tests and simulated terrestrial mission task performance. Aerospace Medicine and Human Performance, 86(11), 982–989. 26564764 10.3357/AMHP.4332.2015

[eph13866-bib-0003] Ade, C. J. , Broxterman, R. M. , Craig, J. C. , Schlup, S. J. , Wilcox, S. L. , Warren, S. , Kuehl, P. , Gude, D. , Jia, C. , & Barstow, T. J. (2016). Prediction of Lunar‐and Martian‐based intra‐and site‐to‐site task performance. Aerospace Medicine and Human Performance, 87(4), 367–374. 27026120 10.3357/AMHP.4399.2016

[eph13866-bib-0004] Ahmadi, M. , Nobari, H. , Ramirez‐Campillo, R. , Pérez‐Gómez, J. , Ribeiro, A. L. D. A. , & Martínez‐Rodríguez, A. (2021). Effects of plyometric jump training in sand or rigid surface on jump‐related biomechanical variables and physical fitness in female volleyball players. International Journal of Environmental Research and Public Health, 18(24), 13093. 34948702 10.3390/ijerph182413093 PMC8701300

[eph13866-bib-0005] Alexander, A. M. , Sutterfield, S. L. , Kriss, K. N. , Hammer, S. M. , Didier, K. D. , Cauldwell, J. T. , Dzewaltowski, A. C. , Barstow, T. J. , & Ade, C. J. (2019). Prediction of emergency capsule egress performance. Aerospace Medicine and Human Performance, 90(9), 782–787. 31426893 10.3357/AMHP.5307.2019

[eph13866-bib-0006] Attias, J. , Philip, A. C. , Waldie, J. , Russomano, T. , Simon, N. E. , & David, A. G. (2017). The Gravity‐Loading countermeasure Skinsuit (GLCS) and its effect upon aerobic exercise performance. Acta Astronautica, 132, 111–116.

[eph13866-bib-0007] Blottner, D. , Hastermann, M. , Weber, R. , Lenz, R. , Gambara, G. , Limper, U. , Rittweger, J. , Bosutti, A. , Degens, H. , & Salanova, M. (2019). Reactive jumps preserve skeletal muscle structure, phenotype, and myofiber oxidative capacity in bed rest. Frontiers in Physiology, 10, 1527. 32009969 10.3389/fphys.2019.01527 PMC6974579

[eph13866-bib-0008] Burnley, M. , & Jones, A. M. (2007). Oxygen uptake kinetics as a determinant of sports performance. European Journal of Sport Science, 7(2), 63–79.

[eph13866-bib-0009] Cavanagh, P. , Genc, K. , Gopalakrishnan, R. , Kuklis, M. , Maender, C. , & Rice, A. (2010). Foot forces during typical days on the international space station. Journal of Biomechanics, 43(11), 2182–2188. 20462584 10.1016/j.jbiomech.2010.03.044

[eph13866-bib-0010] Cavanagh, P. R. , Rice, A. J. , Licata, A. A. , Kuklis, M. M. , Novotny, S. C. , Genc, K. O. , Englehaupt, R. K. , & Hanson, A. M. (2013). A novel lunar bed rest analogue. Aviation Space and Environmental Medicine, 84(11), 1191–1195. 24279234 10.3357/asem.3472.2013

[eph13866-bib-0011] Comfort, P. , Mcmahon, J. , Jones, P. , Cuthbert, M. , Kendall, K. , Lake, J. , & Haff, G. G. (2021). Effects of spaceflight on musculoskeletal health: A systematic review and meta‐analysis, considerations for interplanetary travel. Sports Medicine, 51(10), 2097–2114. 34115344 10.1007/s40279-021-01496-9 PMC8449769

[eph13866-bib-0012] Devita, P. , & Skelly, W. A. (1992). Effect of landing stiffness on joint kinetics and energetics in the lower extremity. Medicine and Science in Sports and Exercise, 24(1), 108–115. 1548984

[eph13866-bib-0013] Dillon, H. T. , Dausin, C. , Claessen, G. , Lindqvist, A. , Mitchell, A. , Wright, L. , Willems, R. , La Gerche, A. , & Howden, E. J. (2021). The effect of posture on maximal oxygen uptake in active healthy individuals. European Journal of Applied Physiology, 121(5), 1487–1498. 33638017 10.1007/s00421-021-04630-7

[eph13866-bib-0014] English, K. L. , Downs, M. , Goetchius, E. , Buxton, R. , Ryder, J. W. , Ploutz‐Snyder, R. , Guilliams, M. , Scott, J. M. , & Ploutz‐Snyder, L. L. (2020). High intensity training during spaceflight: Results from the NASA Sprint Study. Nature Partner Journals Microgravity, 6(1), 21. 10.1038/s41526-020-00111-x PMC7434884 32864428

[eph13866-bib-0015] Flanagan, E. P. , & Comyns, T. M. (2008). The use of contact time and the reactive strength index to optimize fast stretch‐shortening cycle training. Strength & Conditioning Journal, 30, 32–38.

[eph13866-bib-0016] Frost, H. M. (2003). Bone's mechanostat: A 2003 update. The Anatomical Record Part A: Discoveries in Molecular, Cellular, and Evolutionary Biology, 275, 1081–1101. 14613308 10.1002/ar.a.10119

[eph13866-bib-0017] Gaskill, S. E. , Ruby, B. C. , Walker, A. J. , Sanchez, O. A. , Serfass, R. C. , & Leon, A. S. (2001). Validity and reliability of combining three methods to determine ventilatory threshold. Medicine & Science in Sports & Exercise, 33, 1841–1848. 11689733 10.1097/00005768-200111000-00007

[eph13866-bib-0018] Goodwin, M. L. , Harris, J. E. , Hernández, A. , & Gladden, L. B. (2007). Blood lactate measurements and analysis during exercise: A guide for clinicians. Journal of Diabetes Science and Technology, 1(4), 558–569. 19885119 10.1177/193229680700100414 PMC2769631

[eph13866-bib-0019] Gordon, D. , Wightman, S. , Basevitch, I. , Johnstone, J. , Espejo‐Sanchez, C. , Beckford, C. , Boal, M. , Scruton, A. , Ferrandino, M. , & Merzbach, V. (2017). Physiological and training characteristics of recreational marathon runners. Open access journal of sports medicine, 8, 231–241. 29200895 10.2147/OAJSM.S141657 PMC5703178

[eph13866-bib-0020] Gruber, M. , Kramer, A. , Mulder, E. , & Rittweger, J. (2019). The importance of impact loading and the stretch shortening cycle for spaceflight countermeasures. Frontiers in physiology, 10, 311. 30967797 10.3389/fphys.2019.00311 PMC6438856

[eph13866-bib-0021] Hackney, K. J. , Scott, J. M. , Hanson, A. M. , English, K. L. , Downs, M. E. , & Ploutz‐Snyder, L. L. (2015). The astronaut‐athlete: Optimizing human performance in space. The Journal of Strength & Conditioning Research, 29, 3531–3545. 26595138 10.1519/JSC.0000000000001191

[eph13866-bib-0022] Harper Jr, R. P. , & Cooper, G. E. (1986). Handling qualities and pilot evaluation. Journal of Guidance, Control, and Dynamics, 9(5), 515–529.

[eph13866-bib-0023] Heuberger, J. A. , Gal, P. , Stuurman, F. E. , De Muinck Keizer, W. A. , Mejia Miranda, Y. , & Cohen, A. F. (2018). Repeatability and predictive value of lactate threshold concepts in endurance sports. PLoS ONE, 13(11), e0206846. 30427900 10.1371/journal.pone.0206846 PMC6235347

[eph13866-bib-0024] Hughes, L. , Hackney, K. J. , & Patterson, S. D. (2022). Optimization of Exercise Countermeasures to Spaceflight Using Blood Flow Restriction. Aerospace Medicine and Human Performance, 93(1), 32–45. 35063054 10.3357/AMHP.5855.2021

[eph13866-bib-0025] Kassiano, W. , Costa, B. , Nunes, J. P. , Ribeiro, A. S. , Schoenfeld, B. J. , & Cyrino, E. S. (2023). Which ROMs lead to Rome? A systematic review of the effects of range of motion on muscle hypertrophy. The Journal of Strength & Conditioning Research, 37, 1135–1144. 36662126 10.1519/JSC.0000000000004415

[eph13866-bib-0026] Ko, F. C. , Mortreux, M. , Riveros, D. , Nagy, J. A. , Rutkove, S. B. , & Bouxsein, M. L. (2020). Dose‐dependent skeletal deficits due to varied reductions in mechanical loading in rats. Nature Partner Journals Microgravity, 6(1), 15. 10.1038/s41526-020-0105-0 PMC7235020 32435691

[eph13866-bib-0027] Kohrt, W. M. , Barry, D. W. , & Schwartz, R. S. (2009). Muscle forces or gravity: What predominates mechanical loading on bone? Medicine and Science in Sports and Exercise, 41(11), 2050–2055. 19812511 10.1249/MSS.0b013e3181a8c717 PMC3037021

[eph13866-bib-0028] Korth, D. W. (2015). Exercise Countermeasure Hardware Evolution on ISS: The First Decade. Aerospace Medicine and Human Performance, 86, (12:Supplement), A7–A13. 26630190 10.3357/AMHP.EC02.2015

[eph13866-bib-0029] Kramer, A. , Gollhofer, A. , Armbrecht, G. , Felsenberg, D. , & Gruber, M. (2017). How to prevent the detrimental effects of two months of bed‐rest on muscle, bone and cardiovascular system: An RCT. Scientific Reports, 7(1), 1–10. 29030644 10.1038/s41598-017-13659-8 PMC5640633

[eph13866-bib-0030] Kramer, A. , Kümmel, J. , Mulder, E. , Gollhofer, A. , Frings‐Meuthen, P. , & Gruber, M. (2017). High‐intensity jump training is tolerated during 60 days of bed rest and is very effective in preserving leg power and lean body mass: An overview of the cologne RSL study. PLoS ONE, 12(1), e0169793. 28081223 10.1371/journal.pone.0169793 PMC5231329

[eph13866-bib-0031] Kramer, A. , Poppendieker, T. , & Gruber, M. (2019). Suitability of jumps as a form of high‐intensity interval training: Effect of rest duration on oxygen uptake, heart rate and blood lactate. European Journal of Applied Physiology, 119(5), 1149–1156. 30783734 10.1007/s00421-019-04105-w

[eph13866-bib-0032] Laws, J. , Caplan, N. , Bruce, C. , Mcgrogan, C. , Lindsay, K. , Wild, B. , Debuse, D. , Wotring, V. , & Winnard, A. (2020). Systematic review of the technical and physiological constraints of the Orion Multi‐Purpose Crew Vehicle that affect the capability of astronauts to exercise effectively during spaceflight. Acta Astronautica, 170, 665–677.

[eph13866-bib-0033] Leblanc, A. , Lin, C. , Shackelford, L. , Sinitsyn, V. , Evans, H. , Belichenko, O. , Schenkman, B. , Kozlovskaya, I. , Oganov, V. , & Bakulin, A. (2000). Muscle volume, MRI relaxation times (T2), and body composition after spaceflight. Journal of Applied Physiology, 89(6), 2158–2164. 11090562 10.1152/jappl.2000.89.6.2158

[eph13866-bib-0034] Leblanc, A. , Rowe, R. , Schneider, V. , Evans, H. , & Hedrick, T. (1995). Regional muscle loss after short duration spaceflight. Aviation Space and Environmental Medicine, 66, 1151–1554. 8747608

[eph13866-bib-0035] Lee, S. , Feiveson, A. H. , Stein, S. , Stenger, M. B. , & Platts, S. H. (2015). Orthostatic intolerance after ISS and space shuttle missions. Aerospace Medicine and Human Performance, 86, (12:Supplement), A54–A67. 26630196 10.3357/AMHP.EC08.2015

[eph13866-bib-0036] Lee, S. , Moore, A. D. , Everett, M. E. , Stenger, M. B. , & Platts, S. H. (2010). Aerobic exercise deconditioning and countermeasures during bed rest. Aviation, Space, and Environmental Medicine, 81(1), 52–63. 20058738 10.3357/asem.2474.2010

[eph13866-bib-0037] Lyons, T. S. , Reason, K. W. , Tolusso, D. V. , & Weddle, A. S. (2023). Effects of Different Surfaces on Metabolic Cost During Repetitive Jumping: A Pilot Study. International Journal of Exercise Science, 16, 866. 37635918 10.70252/MBDQ5711 PMC10449323

[eph13866-bib-0038] Macinnis, M. J. , & Gibala, M. J. (2017). Physiological adaptations to interval training and the role of exercise intensity. The Journal of Physiology, 595(9), 2915–2930. 27748956 10.1113/JP273196 PMC5407969

[eph13866-bib-0039] Markovic, G. , & Mikulic, P. (2010). Neuro‐musculoskeletal and performance adaptations to lower‐extremity plyometric training. Sports Medicine, 40(10), 859–895. 20836583 10.2165/11318370-000000000-00000

[eph13866-bib-0040] Maturana, F. M. , Schellhorn, P. , Erz, G. , Burgstahler, C. , Widmann, M. , Munz, B. , Soares, R. N. , Murias, J. M. , Thiel, A. , & Nieß, A. M. (2021). Individual cardiovascular responsiveness to work‐matched exercise within the moderate‐and severe‐intensity domains. European Journal of Applied Physiology, 121(7), 2039–2059. 33811557 10.1007/s00421-021-04676-7 PMC8192395

[eph13866-bib-0041] Maynard, C. , Zumbado, F. , Newby, N. , Humphreys, B. T. , & Downs, M. E. (2018). Miniature Exercise Device‐2 (MED‐2): Preliminary ISS Evaluation Results for a Compact Motorized Resistive and Aerobic Rowing Exercise Device. International Space Station Research & Development Conference (ISSR&D 2018).

[eph13866-bib-0042] Messonnier, L. A. , Emhoff, C.‐A. W. , Fattor, J. A. , Horning, M. A. , Carlson, T. J. , & Brooks, G. A. (2013). Lactate kinetics at the lactate threshold in trained and untrained men. Journal of Applied Physiology, 114(11), 1593–1602. 23558389 10.1152/japplphysiol.00043.2013 PMC9094885

[eph13866-bib-0043] Minetti, A. E. , Luciano, F. , Natalucci, V. , & Pavei, G. (2024). Horizontal running inside circular walls of Moon settlements: A comprehensive countermeasure for low‐gravity deconditioning? Royal Society Open Science, 11(5), 231906. 38716331 10.1098/rsos.231906 PMC11076109

[eph13866-bib-0044] Moore, I. S. , Ashford, K. J. , Cross, C. , Hope, J. , Jones, H. S. , & Mccarthy‐Ryan, M. (2019). Humans optimize ground contact time and leg stiffness to minimize the metabolic cost of running. Frontiers in sports and active living, 1, 53. 33344976 10.3389/fspor.2019.00053 PMC7739683

[eph13866-bib-0045] Mortreux, M. , Ko, F. C. , Riveros, D. , Bouxsein, M. L. , & Rutkove, S. B. (2019). Longitudinal time course of muscle impairments during partial weight‐bearing in rats. Nature Partner Journals Microgravity, 5(1), 20. 10.1038/s41526-019-0080-5 PMC6706399 31453318

[eph13866-bib-0046] Muscat, K. M. , Kotrach, H. G. , Wilkinson‐Maitland, C. A. , Schaeffer, M. R. , Mendonca, C. T. , & Jensen, D. (2015). Physiological and perceptual responses to incremental exercise testing in healthy men: Effect of exercise test modality. Applied Physiology, Nutrition, and Metabolism, 40(11), 1199–1209. 10.1139/apnm-2015-0179 26501683

[eph13866-bib-0047] NASA . (2022). *Evidence Report: Risk of Injury and Compromised Performance due to EVA Operations* [Online]. https://ntrs.nasa.gov/citations/20220004017 [Accessed]

[eph13866-bib-0048] Niemeyer, M. , Bergmann, T. G. , & Beneke, R. (2020). Oxygen uptake plateau: Calculation artifact or physiological reality? European Journal of Applied Physiology, 120(1), 231–242. 31748882 10.1007/s00421-019-04267-7

[eph13866-bib-0049] Norcross, J. , Stroud, L. C. , Schaffner, G. , Glass, B. J. , Lee, P. C. , Jones, J. A. , & Gernhardt, M. L. (2008). The effects of terrain and navigation on human extravehicular activity walkback performance on the moon. 79th Annual Scientific Meeting of the Aerospace Medical Association.

[eph13866-bib-0050] Norcross, J. R. , Lee, L. R. , Clowers, K. G. , Morency, R. M. , Desantis, L. , De Witt, J. K. , Jones, J. A. , Vos, J. R. , & Gernhardt, M. L. (2009). Feasibility of performing a suited 10‐km ambulation on the moon‐final report of the EVA walkback test (EWT). (p. 48) Johnson Space Center.

[eph13866-bib-0051] Oxfeldt, M. , Overgaard, K. , Hvid, L. G. , & Dalgas, U. (2019). Effects of plyometric training on jumping, sprint performance, and lower body muscle strength in healthy adults: A systematic review and meta‐analyses. Scandinavian Journal of Medicine & Science in Sports, 29, 1453–1465. 31136014 10.1111/sms.13487

[eph13866-bib-0052] Petersen, N. , Jaekel, P. , Rosenberger, A. , Weber, T. , Scott, J. , Castrucci, F. , Lambrecht, G. , Ploutz‐Snyder, L. , Damann, V. , Kozlovskaya, I. , & Mester, J. (2016). Exercise in space: The European Space Agency approach to in‐flight exercise countermeasures for long‐duration missions on ISS. Extreme Physiology & Medicine, 5(1), 9. 27489615 10.1186/s13728-016-0050-4 PMC4971634

[eph13866-bib-0053] Price, P. D. , Kennett, J. E. , Scott, J. P. , Green, D. A. , & Cleather, D. J. (2024). Landing Style Influences Peak ‘ground’ Reaction Forces During Repeated Jumping Using a Supine Jump Sled in Microgravity. Microgravity Science and Technology, 36(3), 1–10.

[eph13866-bib-0054] Ramírez‐Campillo, R. , Álvarez, C. , Henríquez‐Olguín, C. , Baez, E. B. , Martínez, C. , Andrade, D. C. , & Izquierdo, M. (2014). Effects of plyometric training on endurance and explosive strength performance in competitive middle‐and long‐distance runners. The Journal of Strength & Conditioning Research, 28, 97–104. 23838975 10.1519/JSC.0b013e3182a1f44c

[eph13866-bib-0055] Ramírez‐Delacruz, M. , Bravo‐Sánchez, A. , Esteban‐García, P. , Jiménez, F. , & Abián‐Vicén, J. (2022). Effects of Plyometric Training on Lower Body Muscle Architecture, Tendon Structure, Stiffness and Physical Performance: A Systematic Review and Meta‐analysis. Sports Medicine‐Open, 8(1), 1–29. 35312884 10.1186/s40798-022-00431-0 PMC8938535

[eph13866-bib-0056] Richter, C. , Braunstein, B. , Winnard, A. , Nasser, M. , & Weber, T. (2017). Human Biomechanical and Cardiopulmonary Responses to Partial Gravity—A Systematic Review. Frontiers in Physiology, 8, 583. 28860998 10.3389/fphys.2017.00583 PMC5559498

[eph13866-bib-0057] Ritzmann, R. , Freyler, K. , Kümmel, J. , Gruber, M. , Belavy, D. L. , Felsenberg, D. , Gollhofer, A. , Kramer, A. , & Ambrecht, G. (2018). High intensity jump exercise preserves posture control, gait, and functional mobility during 60 days of bed‐rest: An RCT including 90 days of follow‐up. Frontiers in Physiology, 9, 1713. 30559676 10.3389/fphys.2018.01713 PMC6287051

[eph13866-bib-0058] Robergs, R. A. , Dwyer, D. , & Astorino, T. (2010). Recommendations for improved data processing from expired gas analysis indirect calorimetry. Sports medicine, 40(2), 95–111. 20092364 10.2165/11319670-000000000-00000

[eph13866-bib-0059] Rosenblat, M. A. , Perrotta, A. S. , & Vicenzino, B. (2019). Polarized vs. threshold training intensity distribution on endurance sport performance: A systematic review and meta‐analysis of randomized controlled trials. The Journal of Strength & Conditioning Research, 33, 3491–3500. 29863593 10.1519/JSC.0000000000002618

[eph13866-bib-0060] Schoenfeld, B. J. , & Grgic, J. (2020). Effects of range of motion on muscle development during resistance training interventions: A systematic review. SAGE open medicine, 8, 2050312120901559. 32030125 10.1177/2050312120901559 PMC6977096

[eph13866-bib-0061] Scott, J. M. , Feiveson, A. H. , English, K. L. , Spector, E. R. , Sibonga, J. D. , Lichar Dillon, E. , Ploutz‐Snyder, L. , & Everett, M. E. (2023). Effects of exercise countermeasures on multisystem function in long duration spaceflight astronauts. Nature Partner Journals Microgravity, 9(1), 11. 10.1038/s41526-023-00256-5 PMC9898566 36737441

[eph13866-bib-0062] Scott, J. P. R. , Weber, T. , & Green, D. A. (2019a). Editorial: Optimization of Exercise Countermeasures for Human Space Flight‐Lessons From Terrestrial Physiology and Operational Implementation. Frontiers in Physiology, 10, 1567. 31998142 10.3389/fphys.2019.01567 PMC6965165

[eph13866-bib-0063] Scott, J. P. R. , Weber, T. , & Green, D. A. (2019b). Introduction to the Frontiers Research Topic: Optimization of Exercise Countermeasures for Human Space Flight—Lessons From Terrestrial Physiology and Operational Considerations. Frontiers in Physiology, 10, 173. 30899226 10.3389/fphys.2019.00173 PMC6416179

[eph13866-bib-0064] Seiler, S. (2012). Training intensity distribution. Endurance Training Science and Practice. (pp. 29–41) Inigo Mujika SLU.

[eph13866-bib-0065] Stavnichuk, M. , Mikolajewicz, N. , Corlett, T. , Morris, M. , & Komarova, S. V. (2020). A systematic review and meta‐analysis of bone loss in space travelers. Nature Partner Journals Microgravity, 6(1), 13. 10.1038/s41526-020-0103-2 PMC7200725 32411816

[eph13866-bib-0066] Stöggl, T. , & Sperlich, B. (2014). Polarized training has greater impact on key endurance variables than threshold, high intensity, or high volume training. Frontiers in physiology, 5, 33. 24550842 10.3389/fphys.2014.00033 PMC3912323

[eph13866-bib-0067] Stöggl, T. L. , & Sperlich, B. (2015). The training intensity distribution among well‐trained and elite endurance athletes. Frontiers in Physiology, 6, 295. 26578968 10.3389/fphys.2015.00295 PMC4621419

[eph13866-bib-0068] Stojanović, E. , Ristić, V. , Mcmaster, D. T. , & Milanović, Z. (2017). Effect of plyometric training on vertical jump performance in female athletes: A systematic review and meta‐analysis. Sports Medicine, 47(5), 975–986. 27704484 10.1007/s40279-016-0634-6

[eph13866-bib-0069] Strock, N. , Frisco, D. , Dillon, E. , Estep, P. , Norcross, J. , Prejean, B. , Fincke, R. , & Marshall‐Goebel, K. (2023). Evaluation of Aerobic Standards for Lunar Surface Extravehicular Activities. 2023 Human Research Program (HRP) Investigators’ Workshop (IWS).

[eph13866-bib-0070] Swain, P. , Mortreux, M. , Laws, J. M. , Kyriacou, H. , De Martino, E. , Winnard, A. , & Caplan, N. (2022a). Bone deconditioning during partial weight‐bearing in rodents–A systematic review and meta‐analysis. Life Sciences in Space Research, 34, 87–103. 35940692 10.1016/j.lssr.2022.07.003

[eph13866-bib-0071] Swain, P. , Mortreux, M. , Laws, J. M. , Kyriacou, H. , De Martino, E. , Winnard, A. , & Caplan, N. (2022b). Skeletal muscle deconditioning during partial weight‐bearing in rodents–A systematic review and meta‐analysis. Life Sciences in Space Research, 34, 68–86. 35940691 10.1016/j.lssr.2022.06.007

[eph13866-bib-0072] Tays, G. D. , Hupfeld, K. E. , Mcgregor, H. R. , Salazar, A. P. , De Dios, Y. E. , Beltran, N. E. , Reuter‐Lorenz, P. A. , Kofman, I. S. , Wood, S. J. , & Bloomberg, J. J. (2021). The effects of long duration spaceflight on sensorimotor control and cognition. Frontiers in Neural Circuits, 15, 723504. 34764856 10.3389/fncir.2021.723504 PMC8577506

[eph13866-bib-0073] Teoh, C. E. (2018). Effects of Plyometric Training on Cardiovascular Fitness, Core Stability, and Body Composition. Tunku Abdul Rahman University College.

[eph13866-bib-0074] Wagner, J. , Niemeyer, M. , Infanger, D. , Hinrichs, T. , Streese, L. , Hanssen, H. , Myers, J. , Schmidt‐Trucksäss, A. , & Knaier, R. (2020). New data‐based cutoffs for maximal exercise criteria across the lifespan. Medicine & Science in Sports & Exercise, 52, 1915–1923. 32224715 10.1249/MSS.0000000000002344

[eph13866-bib-0075] Weber, T. , Green, D. A. , Attias, J. , Sies, W. , Frechette, A. , Braunstein, B. , & Rittweger, J. (2019). Hopping in hypogravity‐A rationale for a plyometric exercise countermeasure in planetary exploration missions. PLoS ONE, 14(2), e0211263. 30759113 10.1371/journal.pone.0211263 PMC6373893

[eph13866-bib-0076] Wehrle, A. , Waibel, S. , Gollhofer, A. , & Roecker, K. (2021). Power output and efficiency during supine, recumbent, and upright cycle ergometry. Frontiers in Sports and Active Living, 3, 667564. 34179774 10.3389/fspor.2021.667564 PMC8222662

[eph13866-bib-0077] Whittle, R. S. , Keller, N. , Hall, E. A. , Vellore, H. S. , Stapleton, L. M. , Findlay, K. H. , Dunbar, B. J. , & Diaz‐Artiles, A. (2022). Gravitational Dose‐Response Curves for Acute Cardiovascular Hemodynamics and Autonomic Responses in a Tilt Paradigm. Journal of the American Heart Association, 11(14), e024175. 35861832 10.1161/JAHA.121.024175 PMC9707822

[eph13866-bib-0078] Wilson, G. J. , Elliott, B. C. , & Wood, G. A. (1991). The effect on performance of imposing a delay during a stretch‐shorten cycle movement. Medicine and Science in Sports and Exercise, 23(3), 364–370. 2020276

[eph13866-bib-0079] WMA . (2013). World Medical Association Declaration of Helsinki: Ethical principles for medical research involving human subjects. Journal of the American Medical Association, 310(20), 2191. 24141714 10.1001/jama.2013.281053

[eph13866-bib-0080] Yarmanova, E. N. , Kozlovskaya, I. B. , Khimoroda, N. , & Fomina, E. V. (2015). Evolution of Russian microgravity countermeasures. Aerospace medicine and human performance, 86, (12:Supplement), A32–A37. 26630193 10.3357/AMHP.EC05.2015

[eph13866-bib-0081] Zwiren, L. D. , Freedson, P. S. , Ward, A. , Wilke, S. , & Rippe, J. M. (1991). Estimation of VO2max: A comparative analysis of five exercise tests. Research Quarterly for Exercise and Sport, 62(1), 73–78. 2028096 10.1080/02701367.1991.10607521

