# Supplementary material for: Jumping on the moon as a potential exercise countermeasure
Source: Exp Physiol. 2025 May 11;111(9):4073–88. doi: 10.1113/EP092155 (PMC13394199; doi:10.1113/EP092155)
Supplement: Supplementary file 1 [file EPH-111-4073-s002.docx]

**Supporting Information - Figures**

**Manuscript Title:** Jumping on the Moon as a Potential Exercise Countermeasure

**Supporting Figure 1 (S1)**

Figure 1. Cardiorespiratory responses to incremental jumping in simulated Lunar gravity. Figure displays mean group responses at each jump height (error bars = standard deviation). Abbreviations: VO_2_, volume of oxygen consumption; VCO_2_, volume of carbon dioxide production; VE, minute ventilation; HR, heart rate; RER, respiratory exchange ratio; BF, breathing frequency.

**Supporting Figure 2 (S2)**

Figure 2. Cardiorespiratory responses to incremental jumping in simulated Lunar gravity. Box plots display cohort median (horizontal line), 25-75% quartiles (box boarders) and minima and maxima values (error bars). Filled circles are individual data points. Abbreviations: VO_2_, volume of oxygen consumption; VCO_2_, volume of carbon dioxide production; VE, minute ventilation; HR, heart rate; RER, respiratory exchange ratio; BF, breathing frequency.

**Supporting Figure 3 (S3)**

Figure 3. Relationship (linear) between cardiovascular fitness (VO_2max_) and fractional utilisation of VO_2max_ (%VO_2max_) during jumping at incremental heights in simulated Lunar gravity.

**Supporting Figure 4 (S4)**

Figure 4. Blood lactate response to incremental jumping in simulated Lunar gravity. Box plots display cohort median (horizontal line), 25-75% quartiles (box boarders) and minima and maxima values (error bars). Filled circles are individual data points.

**Supporting Figure 5 (S5)**

Figure 5. Relationship (linear) between blood lactate concentrations and cardiovascular fitness (VO_2max_) during jumping at incremental heights in simulated Lunar gravity.

**Supporting Figure 6 (S6)**

Figure 6. Relationship (linear) between blood lactate concentrations and fractional utilization of aerobic capacity (%VO_2max_) during jumping at incremental heights in simulated Lunar gravity.

**Supporting Figure 7 (S7)**

Figure 7. Biomechanical outcomes for jumping in simulated Lunar gravity. Figure displays mean group responses at each jump height (error bars = standard deviation). Abbreviations: vGRF; vertical ground reaction force; BW, bodyweight.

**Supporting Figure 8 (S8)**

Figure 8. Biomechanical outcomes during jump landings/take-offs performed in simulated Lunar gravity. Box plots display cohort median (horizontal line), 25-75% quartiles (box boarders) and minima and maxima values (error bars). Filled circles are individual data points. Abbreviations: vGRF, vertical ground reaction force; BW, bodyweight.

**Supporting Figure 9 (S9)**

Figure 9. Perceptual responses to jumping in simulated Lunar gravity. Figure displays mean group responses at each jump height (error bars = standard deviation). Abbreviations: RPE; rating of perceived exertion.

**Supporting Figure 10 (S10)**

Figure 10. Perceptual responses to jumping at incremental heights in simulated Lunar gravity. Box plots display cohort median (horizontal line), 25-75% quartiles (box boarders) and minima and maxima values (error bars). Filled circles are individual data points. Abbreviations: RPE, rating of perceived exertion.

**Supporting Figure 11 (S11)**

Figure 11. Relationship between peak vertical ground reaction force (vGRF) against contact time during jumping in simulated Lunar gravity.

**Supporting Figure 12 (S12)**

Figure 12. Relationship between jump depth against contact time during jumping in simulated Lunar gravity.

**Supporting Figure 13 (S13)**

Figure 13. Relationship between relative mean breaking force against breaking phase duration during jumping in simulated Lunar gravity.

**Supporting Figure 14 (S14)**

Figure 14. Relationship between relative mean propulsive force and propulsive phase duration during jumping in simulated Lunar gravity.
