# Supplementary material for: Jumping on the moon as a potential exercise countermeasure
Source: Exp Physiol. 2025 May 11;111(9):4073–88. doi: 10.1113/EP092155 (PMC13394199; doi:10.1113/EP092155)
Supplement: Supplementary file 2 [file EPH-111-4073-s004.docx]

**Supporting Table 1.** Biomechanical outcomes (standard deviations) for incremental jumping in simulated Lunar gravity.

| Outcome | Target Jump Height (cm) | | | | | | | | | R^2^ | p |
| --- | --- | --- | --- | --- | --- | --- | --- | --- | --- | --- | --- |
|  | **30** | **35** | **40** | **45** | **50** | **55** | **60** | **65** | **70** |  |  |
| SD.Jump height (cm) | 2 ± 0 | 2 ± 0 | 3 ± 1 | 2 ± 0 | 3 ± 0 | 3 ± 1 | 3 ± 1 | 3 ± 1 | 3 ± 1 | 0.52 | 0.0614 |
| SD.Jump depth (cm) | 3 ± 1 | 2 ± 1 | 2 ± 1 | 2 ± 1 | 3 ± 2 | 3 ± 1 | 3 ± 1 | 3 ± 1 | 2 ± 1 | 0.24 | 0.1760 |
| SD.Jump frequency (jumps/min) | 1 ± 0 | 1 ± 0 | 1 ± 1 | 1 ± 0 | 1 ± 1 | 1 ± 1 | 1 ± 0 | 1 ± 1 | 1 ± 1 | 0.32 | 0.1791 |
| SD.Contact time (s) | 0.05 ± 0.02 | 0.05 ± 0.02 | 0.04 ± 0.01 | 0.04 ± 0.01 | 0.05 ± 0.03 | 0.04 ± 0.01 | 0.05 ± 0.02 | 0.05 ± 0.02 | 0.04 ± 0.02 | 0.35 | 0.1572 |
| SD.Flight time (s) | 0.04 ± 0.01 | 0.03 ± 0.01 | 0.03 ± 0.01 | 0.03 ± 0.01 | 0.03 ± 0.01 | 0.04 ± 0.01 | 0.04 ± 0.01 | 0.04 ± 0.02 | 0.03 ± 0.01 | 0.10 | 0.4066 |
| SD.Breaking phase duration (s) | 0.03 ± 0.01 | 0.03 ± 0.01 | 0.03 ± 0.01 | 0.02 ± 0.01 | 0.03 ±0.02 | 0.03 ± 0.01 | 0.03 ± 0.01 | 0.03 ± 0.01 | 0.03 ± 0.01 | 0.33 | 0.1745 |
| SD.Propulsive phase duration (s) | 0.03 ± 0.01 | 0.03 ± 0.01 | 0.02 ± 0.01 | 0.03 ±0.01 | 0.03 ± 0.01 | 0.03 ± 0.01 | 0.03 ± 0.01 | 0.03 ± 0.02 | 0.03 ± 0.02 | 0.22 | 0.2902 |
| SD.Peak vGRF (% bodyweight) | 9 ± 6 | 10 ± 7 | 10 ± 6 | 11 ± 8 | 12 ± 9 | 14 ± 8 | 17 ± 12 | 13 ± 4 | 12 ± 4 | 0.55 | 0.0347 |
| SD.Mean breaking force (% bodyweight) | 6 ± 4 | 6 ± 4 | 6 ± 3 | 6 ± 4 | 7 ± 5 | 7 ± 4 | 10 ± 6 | 8 ± 3 | 8 ± 4 | 0.55 | 0.0544 |
| SD.Mean propulsive force (% bodyweight) | 6 ± 3 | 6 ± 3 | 6 ± 2 | 6 ± 3 | 7 ± 4 | 7 ± 3 | 9 ± 6 | 8 ± 3 | 8 ± 3 | 0.38 | 0.1407 |

Note: Sample sizes are as follows: 30cm (n = 15), 35cm (n = 17), 40cm (n = 19), 45cm (n = 19), 50cm (n = 19), 55cm (n = 19), 60cm (n = 16), 65cm (n = 12), 70cm (n = 7). Standard deviation (SD) measures reflect the mean SD of the measurement across every jump for the given stage for each participant (i.e., each participant had a SD score for each measurement calculated from every jump completed in the exercise stage). Jump depth reflects negative centre of mass displacement during landing (standing = 0cm). R^2^ and p-values reflect the median individual R^2^ and p-value for the relationship between jump height and given measurement; all were modelled using linear regression. Abbreviations: vGRF, vertical ground reaction force.
